# The effectiveness of COVID-19 vaccines in reducing the incidence, hospitalization, and mortality from COVID-19: A systematic review and meta-analysis

**DOI:** 10.3389/fpubh.2022.873596

**Published:** 2022-08-26

**Authors:** Kazem Rahmani, Rasoul Shavaleh, Mahtab Forouhi, Hamideh Feiz Disfani, Mostafa Kamandi, Rozita Khatamian Oskooi, Molood Foogerdi, Moslem Soltani, Maryam Rahchamani, Mohammad Mohaddespour, Mostafa Dianatinasab

**Affiliations:** ^1^Department of Epidemiology and Biostatistics, School of Public Health, Iran University of Medical Sciences, Tehran, Iran; ^2^Department of Pharmacy, Shahid Behest University of Medical Sciences, Tehran, Iran; ^3^Department of Emergency Medicine, Faculty of Medicine, Mashhad University of Medical Sciences, Mashhad, Iran; ^4^Hematologist-Oncologist, Department of Internal Medicine, Faculty of Medicine, Mashhad University of Medical Sciences, Mashhad, Iran; ^5^Department of Emergency Medicine, Faculty of Medicine, Birgand University of Medical Sciences, Birjand, Iran; ^6^Department of Gastroenterology and Hepatology, School of Medicine, North Khorasan University of Medical Sciences, Bojnurd, Iran; ^7^Department of Internal Medicine, Faculty of Medicine, Tehran University of Medical Sciences, Tehran, Iran; ^8^Department of Complex Genetics and Epidemiology, School of Nutrition and Translational Research in Metabolism, Maastricht University, Maastricht, Netherlands

**Keywords:** SARS-CoV2 infection, vaccination, hospitalization, mortality, COVID-19, effectiveness

## Abstract

**Background:**

Vaccination, one of the most important and effective ways of preventing infectious diseases, has recently been used to control the COVID-19 pandemic. The present meta-analysis study aimed to evaluate the effectiveness of COVID-19 vaccines in reducing the incidence, hospitalization, and mortality from COVID-19.

**Methods:**

A systematic search was performed independently in Scopus, PubMed *via* Medline, ProQuest, and Google Scholar electronic databases as well as preprint servers using the keywords under study. We used random-effect models and the heterogeneity of the studies was assessed using *I*^2^ and χ^2^ statistics. In addition, the Pooled Vaccine Effectiveness (PVE) obtained from the studies was calculated by converting based on the type of outcome.

**Results:**

A total of 54 studies were included in this meta-analysis. The PVE against SARS-COV 2 infection were 71% [odds ratio (OR) = 0.29, 95% confidence intervals (CI): 0.23–0.36] in the first dose and 87% (OR = 0.13, 95% CI: 0.08–0.21) in the second dose. The PVE for preventing hospitalization due to COVID-19 infection was 73% (OR = 0.27, 95% CI: 0.18–0.41) in the first dose and 89% (OR = 0.11, 95% CI: 0.07–0.17) in the second dose. With regard to the type of vaccine, mRNA-1273 and combined studies in the first dose and ChAdOx1 and mRNA-1273 in the second dose had the highest effectiveness in preventing infection. Regarding the COVID-19-related mortality, PVE was 68% (HR = 0.32, 95% CI: 0.23–0.45) in the first dose and 92% (HR = 0.08, 95% CI: 0.02–0.29) in the second dose.

**Conclusion:**

The results of this meta-analysis indicated that vaccination against COVID-19 with BNT162b2 mRNA, mRNA-1273, and ChAdOx1, and also their combination, was associated with a favorable effectiveness against SARS-CoV2 incidence rate, hospitalization, and mortality rate in the first and second doses in different populations. We suggest that to prevent the severe form of the disease in the future, and, in particular, in the coming epidemic picks, vaccination could be the best strategy to prevent the severe form of the disease.

**Systematic review registration:**

PROSPERO International Prospective Register of Systematic Reviews: http://www.crd.york.ac.uk/PROSPERO/, identifier [CRD42021289937].

## Introduction

Over the past years, emerging and re-emerging diseases became public health challenges due to their high morbidity and mortality (1). In December 2019, an outbreak of SARS coronavirus 2 (SARS-CoV-2) was reported in Wuhan, China, and on 11 March 2020, the World Health Organization (WHO) and the CDC (Center for Disease Control and Prevention) introduced it as COVID-19 (2–4). More than 250 million cases are diagnosed with COVID-19 infection worldwide of which more than 5 million are dead. As a result of this pandemic, many strategies are implemented by governments around the world to prevent further infections and control the pandemic (5, 6). The rapid spread of infection among individuals, the lack of symptoms or mild presentation of the infection during the incubation period, and the contagious nature of the disease during the incubation period have made the epidemic tremendously difficult to be controlled (7, 8). Hence, in addition to the defined protocols, most prevention programs were also concentrated on using vaccines against SARS-CoV-2, after few vaccines were licensed for emergency use by many countries (9–11).

To illustrate the safety of COVID-19 vaccines for mass vaccination, clinical trials of manufactured vaccines showed that the effectiveness of Oxford-AstraZeneca (ChAdOx1), Pfizer BioNTech (BNT162b2 mRNA), Moderna (mRNA-1273), and Johnson & Johnson (Ad26.COV2.S) vaccines in preventing infection were 70.4, 95, 94.1, and 66.9%, respectively (12–14). However, it should be noted that clinical trials are conducted under highly controlled conditions on voluntary entry of certain individuals and groups (15), which can be significantly different from the general population (16, 17). Several observational studies were designed and conducted to determine the effectiveness of mass vaccination of COVID-19 among various populations and groups to not only specify the effectiveness of COVID-19 vaccines in real situations but also to compare the incidence of infection, mortality, and hospitalization due to COVID-19 in larger samples and with a longer follow-up (18–20).

In the meantime, considering the valuable evidence obtained from the effectiveness of vaccination in different groups, it seemed necessary to summarize the scattered evidence through meta-analysis studies. Thus, this study aimed to evaluate the effectiveness of COVID-19 vaccines, the incidence of SARS-CoV-2 infection, and the hospitalization and mortality due to COVID-19 after vaccination in observational studies. The findings of the present study will be applicable and valuable for governments, clinicians, public health authorities, and policymakers to design and implement more effective programs for the prevention of COVID-19.

## Materials and methods

We designed this systematic review and meta-analysis according to the Meta-analysis of Observational Studies in Epidemiology checklist (21) and PRISMA (preferred reporting items for systematic reviews and meta-analyses) standards (22), under a registered protocol at the international PROSPERO (Registration Number: CRD42021289937).

### Search strategy

We searched PubMed, Medline, Scopus, ProQuest, and Google Scholar databases as well as the Preprint servers including medRxiv and Research Square to identify the studies related to the keywords selected based on the Medical Subject Headings (MeSH) published until 15 October 2021, with full texts in English, without any restrictions. The search was performed blindly and independently by two researchers (K.R. and R.S.) using the following keywords in the abovementioned databases by combining four sets of related MeSH and Non-MeSH terms: (1) COVID-19; SARS-CoV-2; coronavirus; (2) vaccine; post-vaccination; (3) mortality; hospitalization; readmission; reinfection; morbidity, and (4) breakthrough infections. Any disagreement in the searches between the two researchers was dealt with by other researchers. Duplicates were also identified by title, author's name, and journal name.

### Eligibility criteria

According to the inclusion criteria, observational studies (case-control, negative case-control, case-based cohort, prospective and retrospective cohorts) were published in English that examined the effectiveness, incidence rate of COVID-19, hospitalization rate, and mortality rate after COVID-19 vaccination were suitable to enter into the meta-analysis. Also, the studies that had examined the confirmed cases of SARS-CoV-2 infection based on positive real-time Reverse Transcription Polymerase Chain Reaction (RT-PCR or PCR) tests were included, and antibody-based studies and the ones based on other diagnostic methods were excluded from the review process. In addition, case reports, case series, letters or correspondence, animal studies, and studies with mathematical model analysis [Such as the SIR model (Susceptible, Infected and Recovers model)] were also excluded (flowchart 1). The studies on autoimmune, immunosuppressed, dialysis patients, or the patients with kidney problems and mental disorders in whom the severity of the disease varied were excluded as well (23–27). Also, the studies that lacked an unvaccinated group to compare the results with were not included in the review process. Also, studies on inactive vaccines such as CoronaVac and Covaxin as well as Ad26.COV2.S were not included in the analysis due to a lack of enough evidence on these vaccines.

### Outcomes

The selected outcomes were as follows:

(1) Effectiveness of the vaccines against infection in the subjects studied (the vaccinated groups compared to the unvaccinated ones), as a relative reduction of RT-PCR test confirmed by throat swab, nasal swab, oropharyngeal swab, or saliva and sputum for COVID-19.

(2) Effectiveness of the vaccines against hospitalization of the subjects in the studies as a relative reduction of hospitalization of the individuals whose RT-PCR test was confirmed by taking throat swab, nasal samples, oropharyngeal swab, or saliva and sputum for COVID-19 disease in the vaccinated groups compared with the unvaccinated ones.

(3) Effectiveness of the vaccines against death of the subjects in the studies as a relative reduction in deaths within 40 days after the RT-PCR test was confirmed by throat swab, nasal swab, oropharyngeal swab, and or saliva and sputum for COVID-19 disease in the vaccinated groups compared with the unvaccinated ones (28).

### Data extraction

Two authors extracted the data from the included studies independently. The extracted information contained the author's names, type of vaccine applied, places of study, study design, description of study conditions including study groups, positive SARS-CoV-2 test cases in vaccinated (after the first and second doses) and unvaccinated groups, and cases of death and hospitalization associated with COVID-19 in vaccinated (after the first and second doses) and unvaccinated groups. We also provided a 95% confidence interval for vaccine efficacy for the first and the second doses. Additionally, if the full text of a study was unavailable or if the reported data were missing key information, we contacted the authors by email at least two times, 1 week apart.

The HR of the studies was considered as the risk ratio of the vaccinated to unvaccinated individuals, and in the studies that HR was calculated as the risk ratio of unvaccinated to vaccinated people, it was inversed ($\frac{1}{HR}$) and a 95% confidence interval was calculated. Also, in the studies that mentioned the effectiveness percentage through 1-HR × 100%, the HR and 95% of confidence intervals were converted by calculating 1-($\frac{HR}{100\%}$). The follow-up periods in the studies were considered based on person-day, even in the studies where the follow-up periods were person-week and person-year.

Considering the studies examined, the people who had not taken any vaccines were classified as unvaccinated, and those who were on the ≥7th day after the first dose and ≥5th day after the second dose were classified as partial vaccinated and fully vaccinated, respectively.

### Quality assessment

The quality of the articles was assessed independently by two of the authors (H.F. and M.K.) using the Newcastle Ottawa Scale (NOS) checklist (29). The included studies were evaluated on three broad criteria: (a) appropriation of the study population selection, (b) comparability of the study groups, and (c) ascertainment of the exposure (for cohort studies) or outcome (for case-control studies) of interest. The scoring range of the checklist was 0 (lowest quality) to 9 (highest quality). In the present research, the studies with a total score of ≥7 were considered high quality (Supplementary Tables 1, 2).

### Statistical analysis

A meta-analysis was carried out using a random-effects model and the Mantel–Haenszel weighting method for each study to estimate pooled Odds Ratios (ORs), pooled Hazard Ratios (HR), and pooled Incidence Rate Ratio (IRR), and 95% confidence intervals were calculated for studies with similar effect measured (OR, IRR, or HR).

The heterogeneity of the studies was assessed using the *I*^2^ *and* χ^2^ statistics, according to the results of which *I*^2^ > 50% with *P*-value <0.1 showed the heterogeneity of the studies. Also, sub-group analysis was performed on the partial vaccinated and full vaccinated individuals based on the type of vaccine and the study design. In addition, to calculate the pooled vaccine effectiveness (PVE) obtained from the studies, the following conversions were used: 1-Pooled Odds Ratio × 100%, 1-Pooled Hazard Ratio × 100%, and 1-Pooled Rate Ratio × 100%. *P* < 0.05 was considered statistically significant unless otherwise specified. The statistical analysis was performed using R version 4.1.1 (30) and Metafor Package (31).

### Sensitivity analysis and publication bias

We conducted a sensitivity analysis to investigate the influence of each individual study or group of studies on the overall risk estimate by removing one study or group of studies at a time. Furthermore, potential publication bias was assessed by visual inspection of Begg funnel plots in which the log RRs were plotted against their standard errors (32).

## Results

### Study characteristics

**Figure 1:**
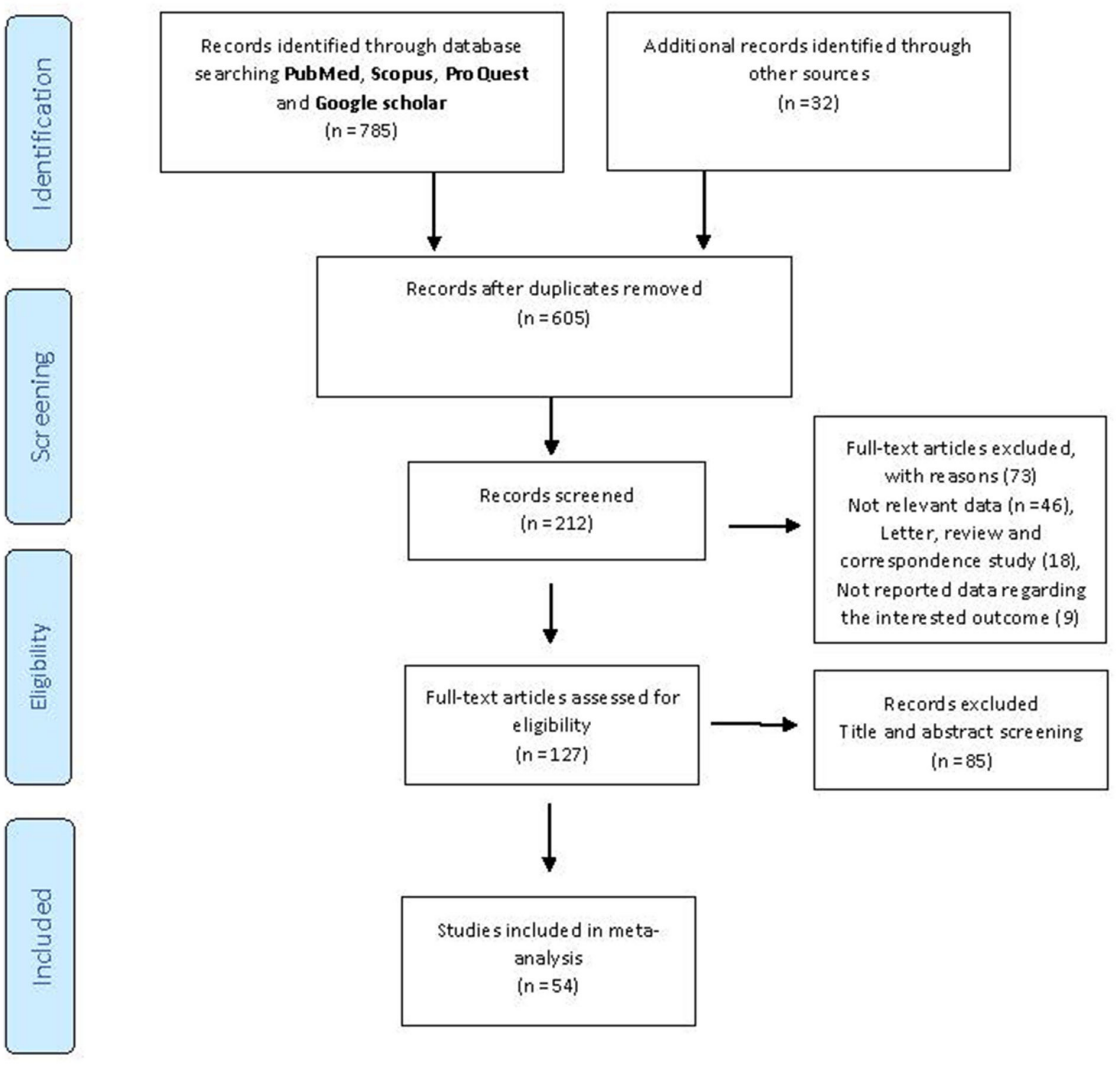
PRISMA flow diagram of the studies included in the meta-analysis.

We initially identified 817 potentially relevant articles from the above-mentioned databases, and 212 records were excluded because they were duplicates. Also, after the title and abstract review, 85 articles were further excluded. Reviewing the full text of the remaining articles, 73 were excluded for the reasons presented in Figure 1. Finally, based on the research strategy, 54 records (11, 18–20, 33–81) on the effectiveness, incidence of SARS-CoV-2 infection, mortality, and hospitalization associated with COVID-19 vaccination were included in the current meta-analysis (the selection procedure is presented in Figure 1). In general, the BNT162b2 mRNA accounted for the most frequent studies on vaccine types (*n* = 37). In terms of location, most of the studies had been conducted in the USA (*n* = 20), UK (*n* = 9), Israel (*n* = 6), and Spain (*n* = 3) (Tables 1–5). All of the included studies were carried out on participants older than 14 years.

**Table 1:** Incidence rate of SARS-CoV-2 infection after the first and second doses in people with a history of COVID-19 vaccination.

**First author**	**Country**	**Type of vaccines**	**Type of study**	**Group of study**	**SARS-COV 2 incidence**									
					**Partial vaccinated**		**Unvaccinated**		Rate ratio^*^	**Full vaccinated**		**Unvaccinated**		Rate ratio^*^
					**Cases**	**Pearson day**	**Cases**	**Pearson day**		**Cases**	**Pearson day**	**Cases**	**Pearson day**	
Noa Dagan (33)	Israel	BNT162b2 mRNA	Prospective cohort study	Age of ≥16 and older	3,533	2,62,180	3,971	2,61,625	0.888	-	-	-	-	-
Hall V. FFPH (34)	UK	BNT162b2 mRNA	Prospective cohort study	Healthcare workers.	71	87,278	977	7,10,587	0.592	9	20,978	977	7,10,587	0.312
Massimo Fabiani (35)	Italy	BNT162b2 mRNA	Retrospective cohort	Healthcare workers.	60	73,914	128	62,331	0.395	0	35,596	11	14,186	0
Eric J. Haas (18)	Israel	BNT162b2 mRNA	Prospective cohort study	Age of 16 and older.	-	-	-	-		6266	20,18,82,183	1,09,876	12,00,76,136	0.034
M. G. Thompson (36)	USA	BNT162b2 mRNA	Prospective cohort study	Health care workers	8	49,516	156	1,27,971	0.133	3	1,20,653	156	1,27,971	0.02
Sara Y. Tartof (19)	USA	BNT162b2 mRNA	Retrospective cohort	Health care workers.	585	1,29,86,040	1,60,280	44,95,93,130	0.126	3414	10,94,42,695	1,60,280	44,95,93,130	0.088
Madhumita Shrotri (37)	UK	BNT162b2 mRNA	Prospective cohort study	Aged 65 years and older	50	17,690	723	3,38,003	1.321	*-*	*-*	*-*	*-*	*-*
Yoel Angel (11)	Israel	BNT162b2 mRNA	Retrospective cohort	Healthcare workers	68	1,17,389	45	15,091	0.194	27	1,68,571	55	25,359	0.074
Colin Pawlowski (38)	USA	BNT162b2 mRNA	Retrospective cohort	Aged ≥18 years.	401	29,42,986	1,232	28,51,069	0.315	82	19,14,500	563	18,28,464	0.139
Gili Regev-Yochay (39)	Israel	BNT162b2 mRNA	Prospective cohort study	Healthcare workers	30	54,832	115	1,99,126	0.947	19	3,29,071	115	1,99,126	0.1
Arjun Puranik (40)	USA	BNT162b2 mRNA	Retrospective cohort	Aged ≥18 years	58	1,80,675	69	1,80,614	0.84	72	23,32,005	321	25,26,895	0.243
Mark A. Katz (41)	Israel	BNT162b2 mRNA	Prospective cohort study	Healthcare workers	*-*	*-*	*-*	*-*		4	68,574	9	10,027	0.065
Carmen Cabezas (42)	Catalonia	BNT162b2 mRNA	Prospective cohort study	Healthcare workers	358	55,28,745	1,961	22,69,003	0.075	222	48,77,162	961	22,69,003	0.107
Aharona Freedman (43)	Israel	BNT162b2 mRNA	Retrospective cohort	Aged ≥16 years	7,166	1,42,89,253	1,33,994	11,97,01,675	0.448	1639	1,40,60,250	95,655	8,95,35,711	0.109
Galia Zacay (44)	Israel	BNT162b2 mRNA	Retrospective cohort	Aged ≥16 years	59	28,727	382	71,797	0.386	15	26,260	382	71,797	0.107
Victoria Jane Hall (34)	UK	BNT162b2 mRNA	Prospective cohort study	Healthcare workers	71	87,278	977	7,10,587	0.592	9	20,978	977	7,10,587	0.312
Hanne-Dorthe Emborg (45)	Denmark	BNT162b2 mRNA	Prospective cohort study	Prioritized risk groups,	610	44,15,441	13,297	5,51,31,206	0.573	304	1,36,12,638	13,297	5,51,31,206	0.093
Jonas Björk (46)	Sweden	BNT162b2 mRNA	Prospective cohort study	Aged 18-64 years,	25	1,02,830	4,155	99,02,620	0.579	8	1,33,616	4,155	99,02,620	0.143
Susana Monge (47)	Spain	BNT162b2 mRNA	Prospective cohort study	Aged ≥65 years	2,690	2,18,621	22	2,128	1.19	885	2,07,774	20	1,997	0.425
Madhumita Shrotri (37)	UK	ChAdOx1	Prospective cohort study	Aged ≥65 years	82	32,672	723	3,38,003	1.173	-	-	-	-	*-*
Subhadeep Ghosh (48)	India	ChAdOx1	Prospective cohort study	Healthcare workers	1,159	4,96,53,918	10,061	10,65,94,492	0.247	2512	5,86,74,639	10,061	10,65,94,492	0.454
M.G. Thompson (36)	USA	mRNA-1273	Prospective cohort study	Health care workers	3	31,231	156	1,27,971	0.079	2	40,394	156	1,27,971	0.041
Colin Pawlowski (38)	USA	mRNA-1273	Retrospective cohort	Aged ≥18 years.	97	9,46,890	303	9,27,716	0.314	7	4,95,550	101	4,78,322	0.067
Arjun Puranik (40)	USA	mRNA-1273	Retrospective cohort	Aged ≥18 years	74	1,80,810	69	1,80,614	1.071	38	22,14,873	321	25,26,895	0.135
Mark G. Thompson (49)	USA	Combination^†^	Prospective cohort study	Healthcare workers	8	41,856	161	1,16,657	0.138	3	78,902	161	1,16,657	0.028
Ashley Fowlkes (50)	USA	Combination^†^	Prospective cohort study	Healthcare workers	-	-	-	-		34	4,54,832	194	1,81,357	0.07
Tara C. Bouton (51)	USA	Combination^†^	Prospective cohort study	Healthcare workers,	29	2,51,790	329	4,06,387	0.142	-	-	-	-	*-*

* Rate Ratio computed.

† BNT162b2 mRNA and mRNA-1273 and ChAdOx1.

**Table 2:** Positive tests for SARS-CoV-2 infection after the first and second doses in people with a history of COVID-19 vaccination.

**First author**	**Country**	**Type of vaccines**	**Type of study**	**Group of study**	**SARS-COV 2 incidence**									
					**Partial vaccinated**		**Unvaccinated**		Rate ratio^*^	**Full vaccinated**		**Unvaccinated**		Rate ratio^*^
					**Cases**	**Pearson day**	**Cases**	**Pearson day**		**Cases**	**Pearson day**	**Cases**	**Pearson day**	
Hall V. FFPH (34)	UK	BNT162b2 mRNA	Prospective cohort study	Healthcare workers.	71	20,641	977	2,683	0.006	9	1,607	977	2,683	0.009
Iván Martínez-Baz (52)	Spain	BNT162b2 mRNA	Prospective cohort study	Aged ≥18 years	90	310	6,980	19,580	0.738	61	491	6,980	19,580	0.256
T. Pilishvili (53)	USA	BNT162b2 mRNA	Case–control study	Healthcare workers	122	1,472	707	3,420	0.347	149	1,472	882	3,420	0.324
Ping Ye, DNP (54)	USA	BNT162b2 mRNA	Retrospective cohort	Nursing home residents	68	86	5	5	0	5	17	5	5	0
Tamara Pilishvili (55)	USA	BNT162b2 mRNA	Case–control study	Health care workers	214	926	340	642	0.267	-	-	-	-	*-*
Iván Martínez-Baz (56)	Spain	BNT162b2 mRNA	Prospective cohort study	Aged ≥18 year	351	2,022	4,811	14,348	0.416	1070	7,972	4,811	14,348	0.307
Sara Carazo (57)	Canada	BNT162b2 mRNA	Case–control study	Healthcare workers	2130	26,719	6,323	24,986	0.256	68	2,022	6,323	24,986	0.103
Carmen Cabezas (42)	Catalonia	BNT162b2 mRNA	Prospective cohort study	Healthcare workers	1607	1,02,161	4,440	1,16,417	0.403	-	-	-	-	*-*
Galia Zacay (44)	Israel	BNT162b2 mRNA	Retrospective cohort	Aged ≥16 years	59	1,445	382	6,286	0.658	16	2,941	382	6,286	0.085
Tariq Azamgarhi (58)	UK	BNT162b2 mRNA	Prospective cohort study	Healthcare workers	23	1,408	26	825	0.51	*-*	*-*	*-*	*-*	-
Jamie Lopez Bernal (59)	UK	BNT162b2 mRNA	Case–control study	Age ≥70 years,	448	1,956	15,287	36,668	0.416	*-*	*-*	*-*	*-*	*-*
Jamie Lopez Bernal (60)	UK	BNT162b2 mRNA	Case–control study	Aged ≥16 years,	43	2,884	4,043	96,371	0.346	122	15,749	4,043	96,371	0.178
T. Pilishvili (53)	USA	mRNA-1273	Case–control study	Health care workers	18	1,472	156	3,420	0.259	18	1,472	190	3,420	0.21
Tamara Pilishvili (55)	USA	mRNA-1273	Case–control study	Health care workers	68	268	340	642	0.302	*-*	*-*	*-*	*-*	
Iván Martínez-Baz (56)	Spain	mRNA-1273	Prospective cohort	Aged ≥18 year	70	517	4,811	14,348	0.31	85	1,127	4,811	14,348	0.162
Sara Carazo (57)	Canada	mRNA-1273	Case–control study	Healthcare workers	110	1,639	6,323	24,986	0.212	2	128	6,323	24,986	0.047
Jamie Lopez Bernal (60)	UK	mRNA-1273	Case control	Aged ≥16 years,	592	25,913	4,043	96,371	0.534	218	8,244	4,043	96,371	0.62
Saurabh Bobdey (61)	India	ChAdOx1	Prospective cohort study	-	27	239	19	94	0.503	67	2,863	19	94	0.095
Iván Martínez-Baz (52)	Spain	ChAdOx1	Prospective cohort study	Aged ≥18 years	99	524	6,980	19,580	0.42	-	-	-	-	*-*
Iván Martínez-Baz (56)	Spain	ChAdOx1	Prospective cohort study	Aged ≥18 year -	302	1,599	4,811	14,348	0.462	272	1,539	4,811	14,348	0.426
Aleena Issac (62)	India	ChAdOx1	Prospective cohort study	Healthcare Workers	-	-	-	-	*-*	16	243	35	80	0.091
Jamie Lopez Bernal (59)	UK	ChAdOx1	Case–control study	Adult age ≥70 years,	396	1,342	15,287	36,668	0.585	-	-	-	-	*-*
Eli S. Rosenberg (63)	USA	Combination^†^	Prospective cohort	Adults aged ≥18	-	-	-	-	*-*	9675	1,01,35,322	38,505	37,42,197	0.092
Maria Elena Flacco (64)	Italy	Combination^†^	Retrospective cohort	Aged ≥18 years.	12	69,539	6,948	1,75,687	0.004	-	-	-	-	*-*
Aaron J. Tande (65)	USA	Combination^†^	Retrospective cohort	Aged ≥18 years	42	3,006	1,436	45,327	0.433	-	-	-	-	*-*
Anoop S. V. Shah (66)	Scotland	Combination^†^	Prospective cohort study	Healthcare workers	1152	1,09,074	3,191	1,44,525	0.473	-	-	-	-	*-*
Kristin L. Andrejko (67)	USA	Combination^†^	Case–control study	Aged ≥18 years	51	150	454	767	0.355	20	106	454	767	0.16
Nathanael Fillmore (68)	USA	Combination^†^	Retrospective cohort	-	-	-	-	-	*-*	1546	3,627	6,326	11,569	0.616
Tara C. Bouton (51)	USA	Combination^†^	Prospective cohort study	Healthcare workers	96	7,109	329	3,481	0.131	17	5,913	329	3,481	0.028
Hannah Chung (69)	Canada	Combination^†^	Case–control study	Aged ≥16 years	2050	21,272	51,220	3,02,761	0.524	73	21,272	51,220	3,02,761	0.017
Alyson Cavanaugh (70)	USA	Combination^†^	Case–control study	Aged ≥18 years	17	56	179	463	0.692	50	219	179	463	0.469

* Odds Ratio computed.

† BNT162b2 mRNA and mRNA-1273 and ChAdOx1.

**Table 3:** COVID-19-related mortality after the first and second doses in people vaccinated with BNT162b2 mRNA.

**First author**	**Vaccine type**	**Country**	**Type of study**	**Group of study**	**SARS-COV 2 incidence**									
					**Partial vaccinated**		**Unvaccinated**		Rate ratio^*^	**Full vaccinated**		**Unvaccinated**		Rate ratio^*^
					**Death**	**Pearson day**	**Death**	**Pearson day**		**Death**	**Pearson day**	**Death**	**Pearson day**	
Noa Dagan (33)	BNT162b2 mRNA	Israel	Prospective cohort study	Age of ≥16 and older	2	2,64,538	6	2,64,479	0.333	9	4,322	32	4,316	0.281
Eric J. Haas (18)	BNT162b2 mRNA	Israel	Prospective cohort study	Age of 16 and older.	-	-	-	-	*-*	138	20,18,82,183	715	12,00,76,136	0.115
Arjun Puranik (40)	BNT162b2 mRNA	USA	Retrospective cohort	Aged ≥18 years	0	1,80,814	0	1,80,819	*-*	0	23,33,860	4	25,37,030	0
Carmen Cabezas (42)	BNT162b2 mRNA	Catalonia	Prospective cohort study	Healthcare workers	153	30,30,779	272	6,39,181	0.119	33	27,45,713	272	6,39,181	0.028
Aharona Freedman (43)	BNT162b2 mRNA	Israel	Retrospective cohort	Aged ≥16 years	178	1,42,89,253	819	11,97,01,675	1.821	61	1,40,60,250	567	8,95,35,711	0.685
Hanne-Dorthe Emborg (45)	BNT162b2 mRNA	Denmark	Prospective cohort study	-	203	45,51,842	445	5,59,10,554	5.603	25	1,37,35,570	445	5,59,10,554	0.229
Arjun Puranik (40)	mRNA-1273	USA	Retrospective cohort	Aged ≥18 years	0	1,80,951	0	1,80,819	*-*	0	22,15,773	4	25,37,030	0
Subhadeep Ghosh (48)	ChAdOx1	India	Prospective cohort study	Healthcare workers	16	4,96,53,918	37	10,65,94,492	0.928	7	5,86,74,639	37	10,65,94,492	0.344
Baltazar Nunes (71)	Combination^†^	Portugal	Prospective cohort study	Aged ≥65 years	11	2,15,60,915	90	5,29,45,805	0.3	14	4,88,53,060	90	5,29,45,805	0.169

* Rate Ratio computed.

† BNT162b2 mRNA and mRNA-1273 and ChAdOx1.

**Table 4:** COVID-19-related hospitalization rate after the first and second doses of vaccinated patients.

**First author**	**Vaccine type**	**Country**	**Type of study**	**Group of study**	**SARS-COV 2 incidence**									
					**Partial vaccinated**		**Unvaccinated**		Odds ratio^*^	**Full vaccinated**		**Unvaccinated**		Odds ratio^*^
					**Cases**	**Total**	**Cases**	**Total**		**Cases**	**Total**	**Cases**	**Total**	
Iván Martínez-Baz (56)	BNT162b2 mRNA	Spain	Prospective cohort study	Aged ≥18 year	6	2,022	214	14,348	0.197	20	7,972	214	14,348	0.166
Wesley H. Self (72)	BNT162b2 mRNA	USA	Case–control study	Among Adults	-	*-*	*-*	*-*	*-*	128	738	1,463	2,362	0.129
Jamie Lopez Bernal (59)	BNT162b2 mRNA	UK	Case–control study	Age ≥70 years,	128	1,400	1,365	8,892	0.555	*-*	*-*	*-*	*-*	*-*
M.G. Thompson (20)	BNT162b2 mRNA	USA	Case–control study	Aged ≥50 years	140	1,444	3,695	20,406	0.486	220	9,848	3,695	20,406	0.103
Iván Martínez-Baz (56)	mRNA-1273	Spain	Prospective cohort study	Aged ≥18 year	2	517	214	14,348	0.256	1	1,127	214	14,348	0.059
M.G. Thompson (20)	mRNA-1273	USA	Case–control study	Aged ≥50 years	91	1,639	3,695	20,406	0.266	145	7,508	3,695	20,406	0.089
Iván Martínez-Baz (56)	ChAdOx1	Spain	Prospective cohort study	Aged ≥18 year	8	1,599	214	14,348	0.332	2	1,539	214	14,348	0.086
Jamie Lopez Bernal (59)	ChAdOx1	UK	Case–control study	Adult age ≥70	9	126	1,365	8,892	0.424	*-*	*-*	*-*	*-*	*-*
Anoop S. V. Shah (66)	Combination^†^	Scotland	Prospective cohort study	Healthcare workers	19	1,11,081	158	1,44,525	0.156	*-*	*-*	*-*	*-*	*-*
Jennifer B. Griffin (73)	Combination^†^	USA	Prospective cohort study	Aged ≥16 years	29	1,431	1,289	30,801	0.474	136	10,895	1,289	30,801	0.289

* Rate Ratio computed.

† BNT162b2 mRNA and mRNA-1273 and ChAdOx1.

**Table 5:** Hazard ratio of SARS-COV 2 infection and COVID-19-related mortality in patients with a history of first- and second-dose vaccination.

**First author**	**Vaccine type**	**Country**	**Type of study**	**Group of study**	**HR SARS-COV 2 infection**						**HR death related to the COVID-19**					
					**Partial vaccinated**			**Full vaccinated**			**Partial vaccinated**			**Full vaccinated**		
					HR^*^	**95% CI**		HR^*^	**95% CI**		HR^*^	**95% CI**		HR^*^	**95% CI**	
Hall V. FFPH (34)	BNT162b2 mRNA	UK	Prospective cohort study	Healthcare workers.	0.3	0.15	0.45	0.15	0.04	0.26	*-*	*-*	*-*	*-*	*-*	*-*
Amadea Britton (74)	BNT162b2 mRNA	USA	Retrospective cohort	-	0.37	0.21	0.67	*-*	*-*	*-*	*-*	*-*	*-*	*-*	*-*	*-*
Adeel A. Butt (75)	BNT162b2 mRNA	Qatar	Prospective cohort study	-	*-*	*-*	*-*	*-*	*-*	*-*	*-*	*-*	*-*	0.35	0.22	0.55
Ioannis Baltas (76)	BNT162b2 mRNA	UK	Case–control study	-	*-*	*-*	*-*	*-*	*-*	*-*	0.34	0.178	0.651			
M. G. Thompson (36)	BNT162b2 mRNA	USA	Prospective cohort study	Healthcare workers	0.2	0.1	0.4	0.07	0.02	0.22	*-*	*-*	*-*	*-*	*-*	*-*
Sara Y. Tartof (19)	BNT162b2 mRNA	USA	Retrospective cohort	Aged ≥12 years.	0.42	0.39	0.46	0.27	0.26	0.28	*-*	*-*	*-*	*-*	*-*	*-*
Madhumita Shrotri (37)	BNT162b2 mRNA	UK	Prospective cohort study	Age of ≥65	0.77	0.37	1.58	*-*	*-*	*-*	*-*	*-*	*-*	*-*	*-*	*-*
Mark A. Katz (41)	BNT162b2 mRNA	Israel	Prospective cohort study	Healthcare workers	*-*	*-*	*-*	0.055	0.018	0.174	*-*	*-*	*-*	*-*	*-*	*-*
Jamie Lopez Bernal (77)	BNT162b2 mRNA	UK	Retrospective cohort	Aged ≥70 years	*-*	*-*	*-*	*-*	*-*	*-*	0.56	0.47	0.68	0.31	0.14	0.69
Ben Glampson (78)	BNT162b2 mRNA	UK	Retrospective cohort	Aged ≥16 years.	0.42	0.36	0.5	*-*	*-*	*-*	*-*	*-*	*-*	*-*	*-*	*-*
Carmen Cabezas (42)	BNT162b2 mRNA	Catalonia	Prospective cohort study	Healthcare workers	0.13	0.11	0.14	0.13	0.11	0.16	0.31	0.26	0.39	0.03	0.02	0.04
Tariq Azamgarhi (58)	BNT162b2 mRNA	UK	Prospective cohort study	Healthcare workers	0.3	0.09	0.94	*-*	*-*	*-*	*-*	*-*	*-*	*-*	*-*	*-*
Jamie Lopez Bernal (59)	BNT162b2 mRNA	UK	Case–control study	Adult age ≥70 years,	*-*	*-*	*-*	*-*	*-*	*-*	0.49	0.38	0.63	*-*	*-*	*-*
Ida Rask Moustsen-Helms (79)	BNT162b2 mRNA	Denmark	Retrospective cohort	Healthcare workers,	0.17	0.04	0.28	0.9	0.82	0.95	*-*	*-*	*-*	*-*	*-*	*-*
Peter Nordstrom (80)	BNT162b2 mRNA	Sweden	Prospective cohort	-	*-*	*-*	*-*	0.22	0.21	0.22	*-*	*-*	*-*	*-*	*-*	*-*
M.G. Thompson (36)	mRNA-1273	USA	Prospective cohort study	Health care workers	0.17	0.05	0.6	0.18	0.04	0.8	*-*	*-*	*-*	*-*	*-*	*-*
Peter Nordstrom (80)	mRNA-1273	Sweden	Prospective cohort	-	*-*	*-*	*-*	0.13	0.12	0.16	*-*	*-*	*-*	*-*	*-*	*-*
Saurabh Bobdey (61)	ChAdOx1	India	Prospective cohort study	-	0.559	0.327	0.954	0.114	0.0763	0.184	*-*	*-*	*-*	*-*	*-*	*-*
Ioannis Baltas (76)	ChAdOx1	UK	Case–control study	-	*-*	*-*	*-*	*-*	*-*	*-*	0.216	0.067	0.696	*-*	*-*	*-*
Madhumita Shrotri (37)	ChAdOx1	UK	Prospective cohort study	Aged ≥65 years	0.95	0.5	1.84	*-*	*-*	*-*	*-*	*-*	*-*	*-*	*-*	*-*
Jamie Lopez Bernal (77)	ChAdOx1	UK	Retrospective cohort	Aged ≥70 years	*-*	*-*	*-*	*-*	*-*	*-*	0.45	0.34	0.59	*-*	*-*	*-*
Ben Glampson (78)	ChAdOx1	UK	Retrospective cohort	Aged ≥16 years.	0.59	0.49	0.71	*-*	*-*	*-*	*-*	*-*	*-*	*-*	*-*	*-*
Peter Nordstrom (80)	ChAdOx1	Sweden	Prospective cohort	-	*-*	*-*	*-*	0.5	0.42	0.59	*-*	*-*	*-*	*-*	*-*	*-*
Mark G. Thompson (49)	Combination^†^	USA	Prospective cohort study	Healthcare workers	0.2	0.1	0.41	0.1	0.03	0.32	*-*	*-*	*-*	*-*	*-*	*-*
Ashley Fowlkes (50)	Combination^†^	USA	Prospective cohort study	Healthcare workers				0.2	0.12	0.31	*-*	*-*	*-*	*-*	*-*	*-*
Sarah E. Waldman (81)	Combination^†^	USA	Retrospective cohort	Age ≥18 year old	0.53	0.4	0.71	0.22	0.12	0.42	*-*	*-*	*-*	*-*	*-*	*-*
Maria Elena Flacco (64)	Combination^†^	Italy	Retrospective cohort	Aged ≥18 years.	0.05	0.04	0.06	0.02	0.01	0.03	0.03	0.01	0.08	0.02	0	0.12
Baltazar Nunes (71)	Combination^†^	Portugal	Prospective cohort study	Aged ≥65 years.	*-*	*-*	*-*	*-*	*-*	*-*	0.23	0.12	0.44	0.04	0.02	0.08

* Hazard Ratio adjusted in each study.

† BNT162b2 mRNA and mRNA-1273 and ChAdOx1.

### Results

#### Effectiveness of vaccines against SARS-CoV-2 infection, hospitalization, and mortality related to the COVID-19 in partial vaccinated individuals

##### SARS-CoV-2 infection

**Figure 2:**
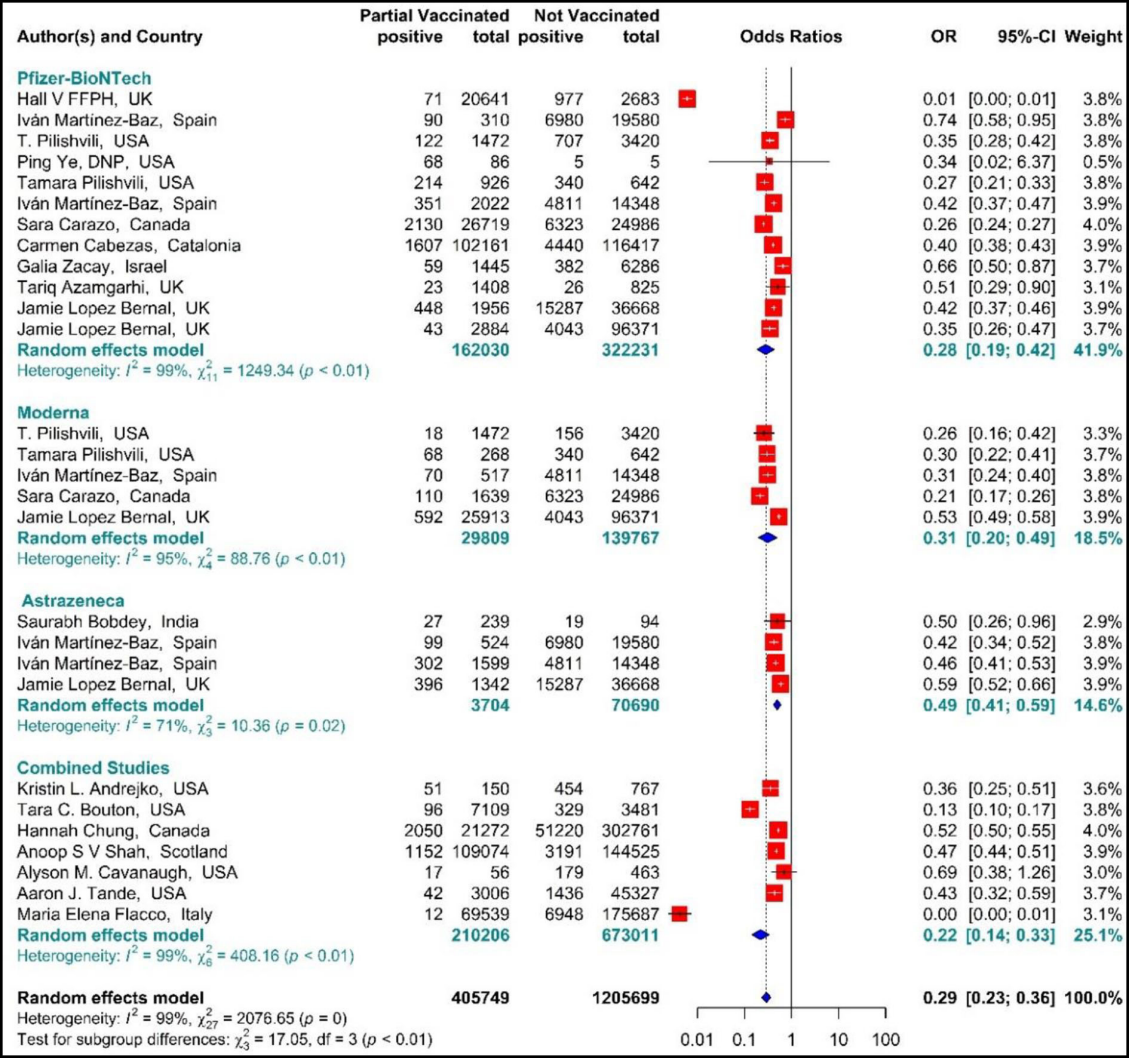
Effectiveness of vaccines against SARS-CoV-2 infection using odds ratio in partial vaccinated individuals.

The results of the forest plot using effect measure pooled OR for the included studies (34, 42, 44, 51–61, 64–67, 69, 70) revealed that the effectiveness of the first dose (partial) of the selected vaccines against SARS-CoV-2 infection was 71% in total (OR = 0.29, 95% CI: 0.23–0.36). This effectiveness varied according to the type of vaccine (*p*−*value*_*subgroup*_ < 0.01); that is, the effectiveness of BNT162b2 mRNA vaccine against SARS-COV 2 infection was 72% (pooled OR = 0.28, 95% CI: 0.19–0.42), the effectiveness of mRNA-1273 vaccine was 69% (pooled OR = 0.31, 95% CI: 0.20–0.49), and that of ChAdOx1 vaccine was 51% (pooled OR = 0.49 95% CI: 0.41–0.59). Furthermore, the combined studies (those who were vaccinated with different types of vaccines) that examined the vaccines (BNT162b2 mRNA, mRNA-1273, ChAdOx1) reported an approximate effectiveness of 78% (pooled OR = 0.22, 95% CI: 0.14–0.33) (Figure 2).

**Figure 3:**
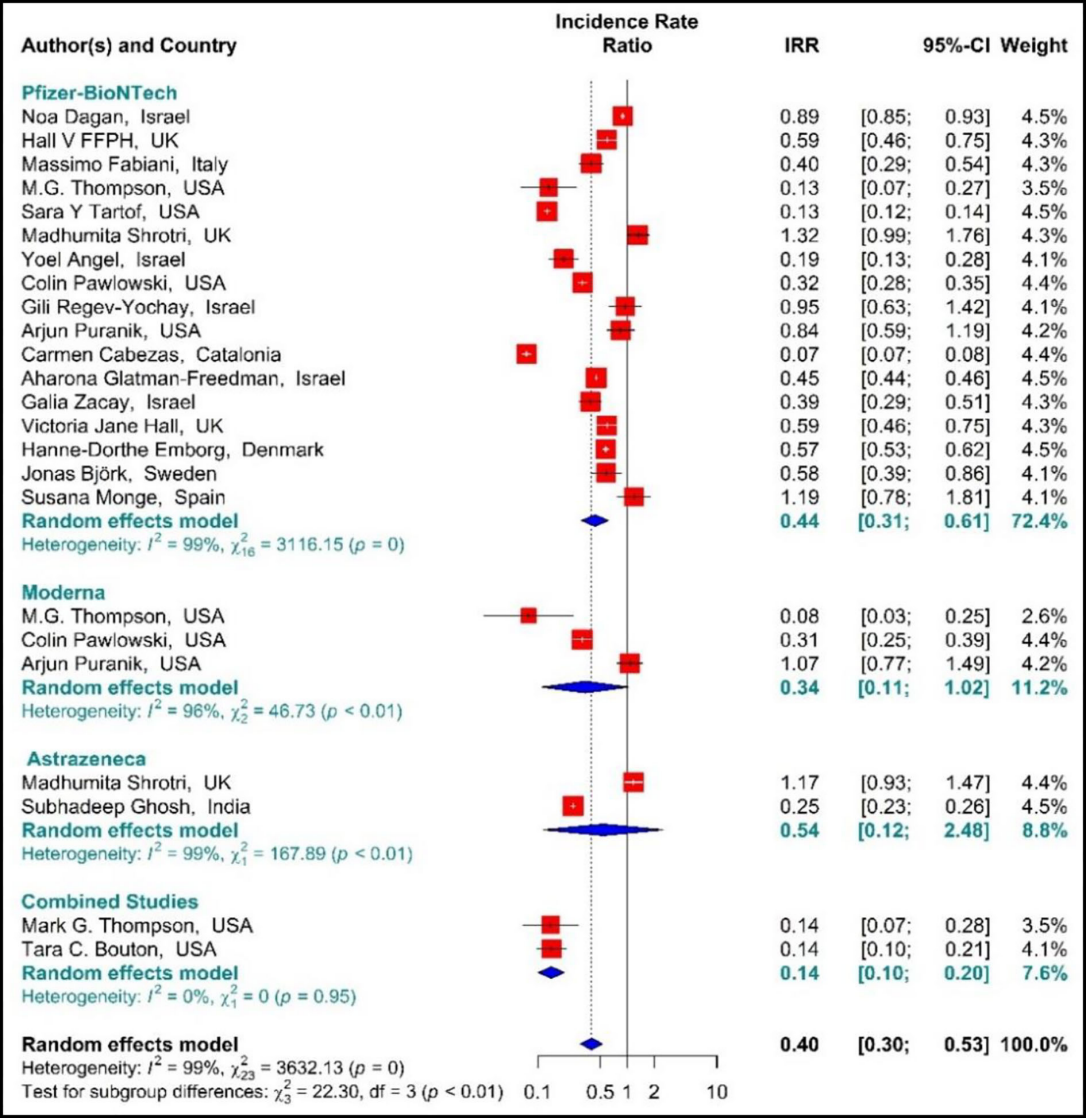
Effectiveness of vaccines against SARS-CoV-2 infection using incidence rate ratio (IRR) in partial vaccinated individuals.

The estimated effectiveness of vaccines against SARS-CoV-2 infection using IRR indicated that the rate of SARS-COV 2 infection in the people vaccinated with BNT162b2 mRNA, mRNA-1273, ChAdOx1, and Combined studies on the first dose was reduced by 60% (IRR = 0.4, 95% CI: 0.30–0.53 (Figure 3). The reduction in SARS-CoV-2 infection rate in the individuals vaccinated with the first dose of BNT162b2 mRNA, mRNA-1273, and ChAdOx1 was 56% (IRR = 0.44, 95% CI: 0.31–0.61), 66% (IRR = 0.34, 95% CI: 0.11–1.02), and 46% (IRR = 0.54, 95% CI: 0.12–2.48), respectively. In the combined studies, the reduction in SARS-CoV-2 infection was 86% (IRR = 0.14, 95% CI: 0.10–0.20). The results of the sub-group analysis in the first dose showed well that there was a significant difference between the effectiveness of different types of vaccines against SARS-COV 2 infection (*p*−*value*_*subgroup*_ < 0.01) (Figure 3).

**Figure 4:**
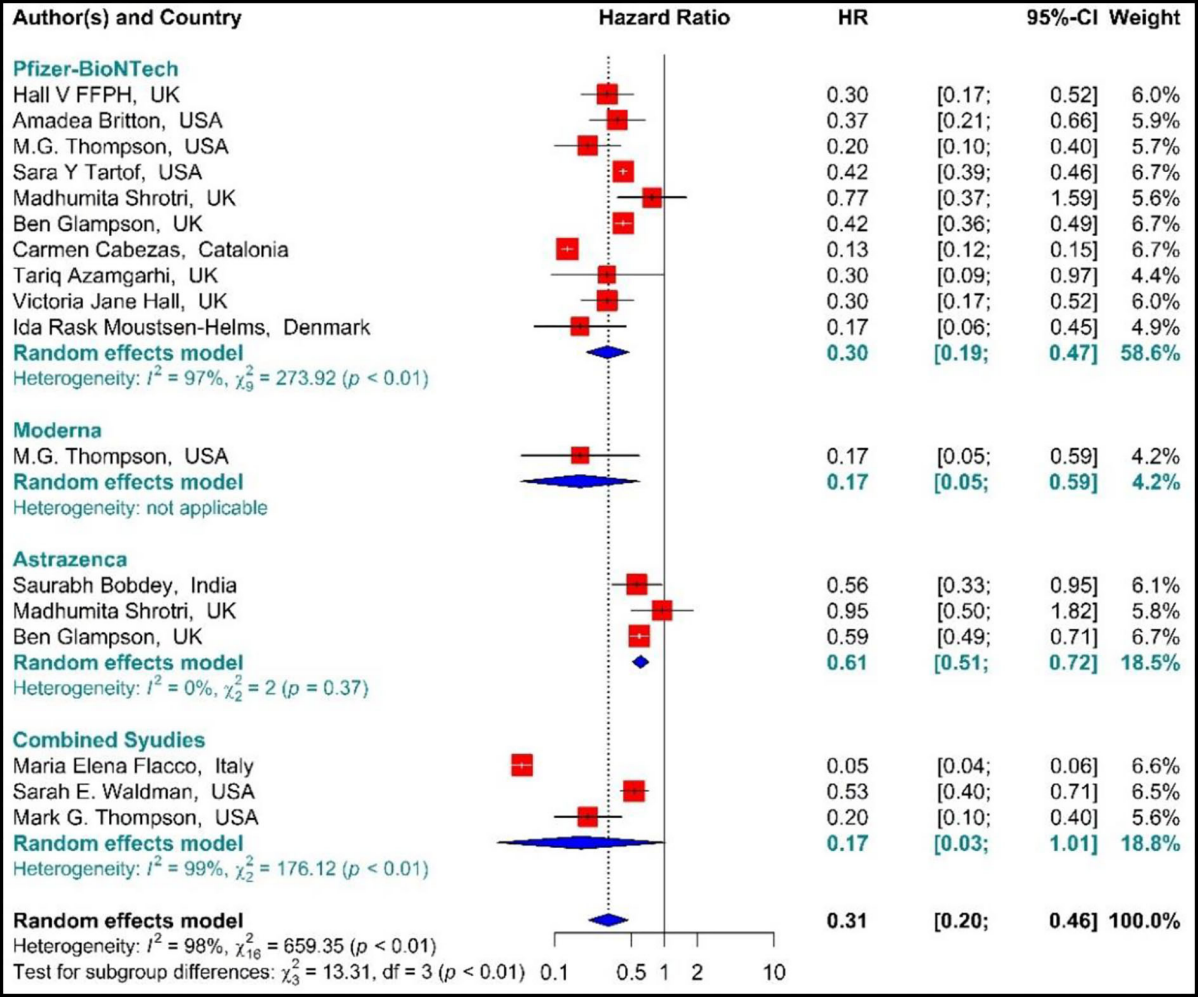
Effectiveness of vaccines against SARS-CoV-2 infection using hazard ratio in partial vaccinated individuals.

Studying the Hazard ratio associated with SARS-CoV-2 infection showed that vaccination with the first dose of BNT162b2 mRNA, mRNA-1273, ChAdOx1, and Combined studies reduced the risk of SARS-CoV-2 infection by 69% (HR = 0.31, 95% CI: 0.20–0.46) (Figure 3) (19, 34, 36, 37, 42, 49, 58, 61, 64, 74, 78, 79, 81). In other words, the first doses of BNT162b2 mRNA, mRNA-1273, and ChAdOx1 vaccines had reduced the SARS-CoV-2 infection by 70% (HR = 0.30, 95% CI: 0.19–0.47), 83% (HR = 0.17, 95% CI: 0.05–0.59), and 39% (HR = 0.61, 95% CI: 0.51–0.72), respectively. On the other hand, the combined studies had reduced the risk of SARS-COV 2 infection by 83% (HR = 0.17, 95% CI: 0.03–1.01). The results of the sub-group analysis on those who received the first dose suggested that there was a difference between the effectiveness of different types of vaccines against SARS-CoV-2 infection (*p*−*value*_*subgroup*_ < 0.01) (Figure 4).

##### Hospitalization

**Figure 5:**
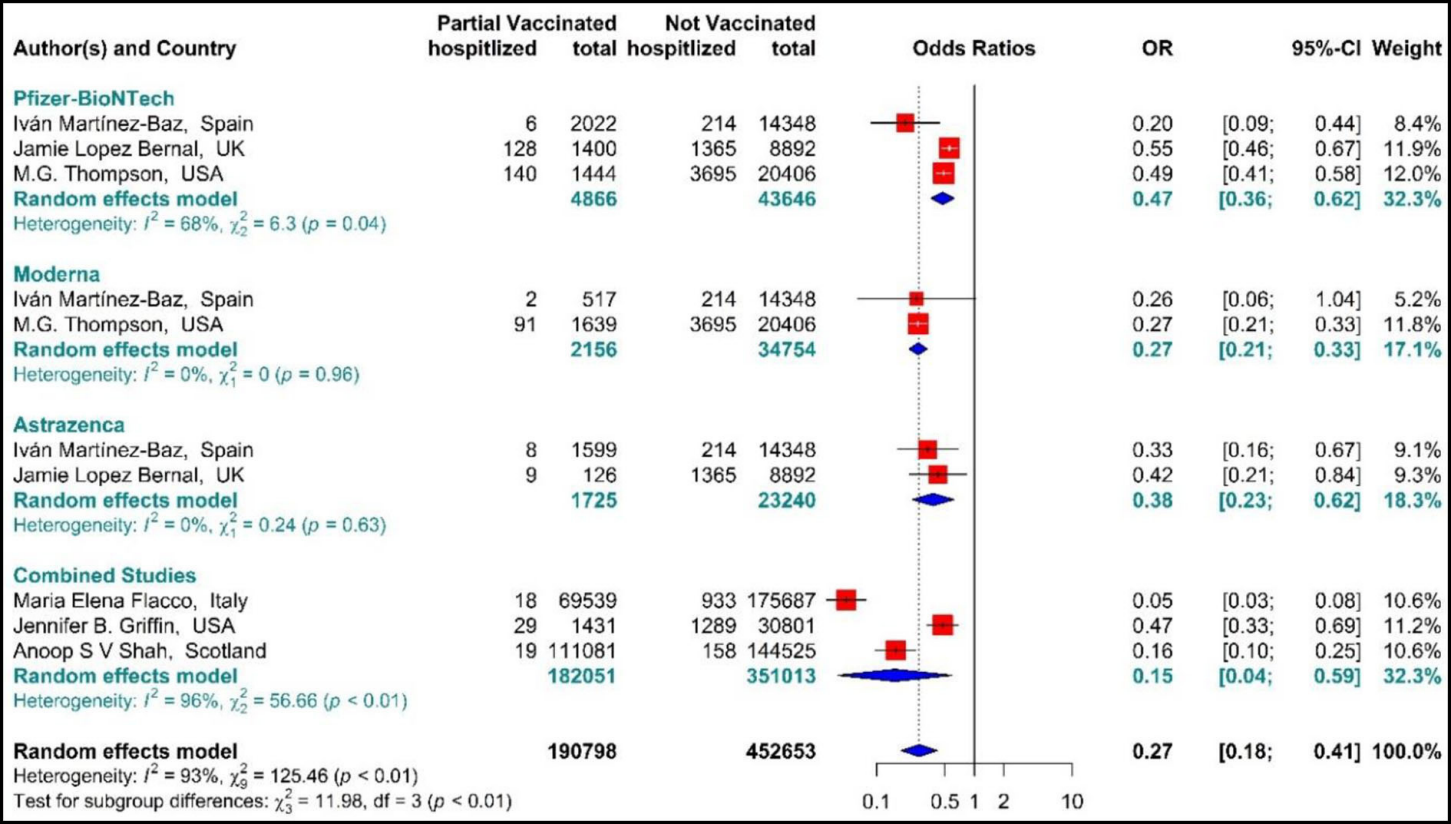
Effectiveness of vaccines against COVID-19-related hospitalization in partial vaccinated individuals.

The total effectiveness of BNT162b2 mRNA, mRNA-1273, and ChAdOx1 vaccines as well as the combined studies in the first dose against COVID-19-related hospitalization was 73% (OR = 0.27, 95% CI: 0.18–0.41) (Figure 5) (20, 56, 59, 64, 66, 73). Considering the type of vaccines, the results of pooled analysis showed that the effectiveness of BNT162b2 mRNA vaccine was 53% (OR = 0.47, 95% CI: 0.36–0.62), that of mRNA-1273 was 73% (OR = 0.27, 95% CI: 0.21–0.33), and the effectiveness of ChAdOx1 vaccine was about 62% (OR = 0.38, 95% CI: 0.23–0.62). In the Combined studies, the pooled efficacy of the vaccines was about 85% (OR = 0.15, 95% CI: 0.04–0.59). The results of the sub-group analysis on the type of vaccines indicated no significant difference between the effectiveness of the vaccines in the first dose against hospitalization with COVID-19 (*p*−*value*_*subgroup*_ < 0.01) (Figure 5).

##### Mortality

**Figure 6:**
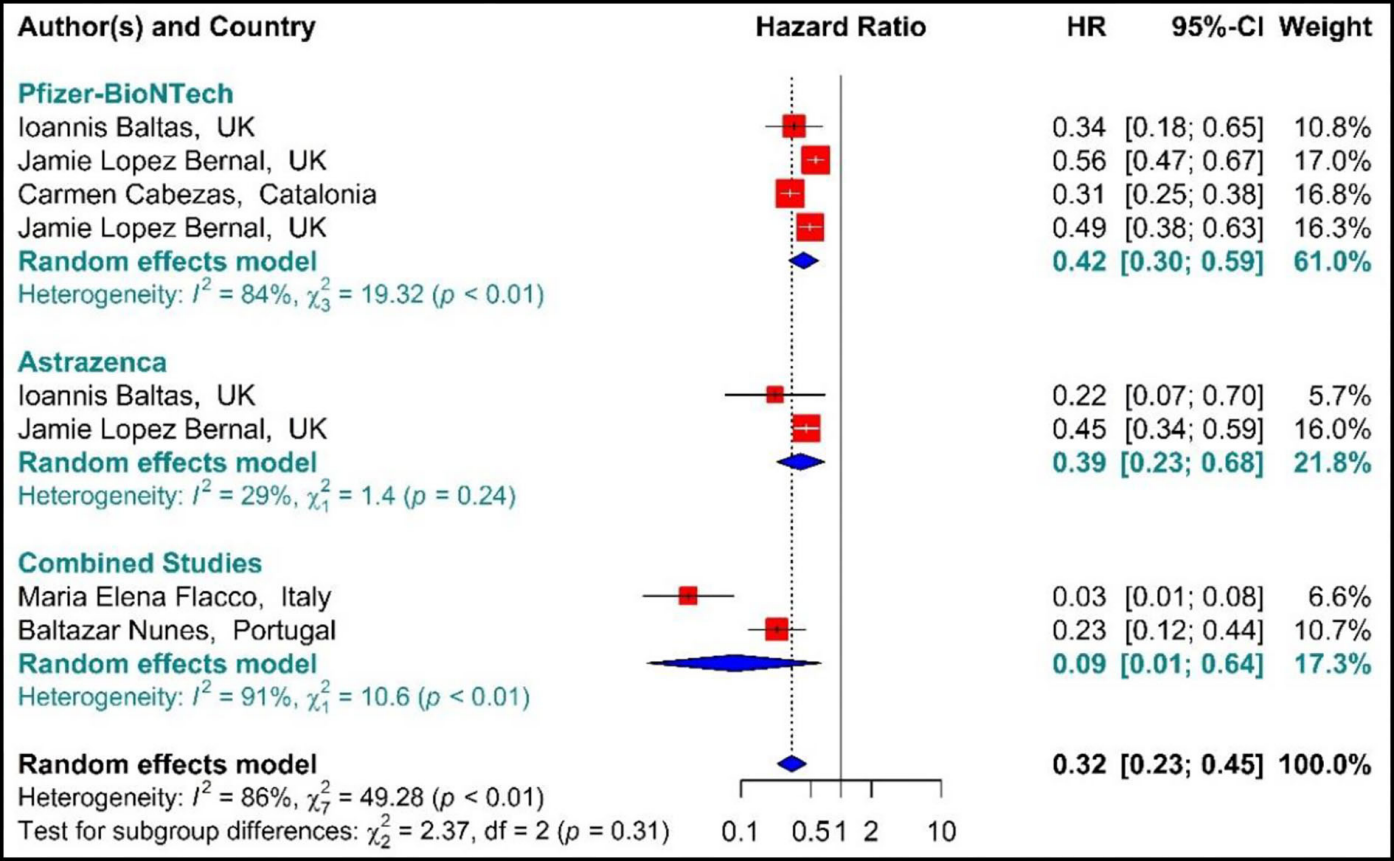
Effectiveness of vaccines against COVID-19-related mortality using hazard ratio in partial vaccinated individuals.

As presented in Figure 6, the COVID-19-associated mortality Hazard ratio in the first-dose vaccinated individuals (42, 59, 64, 71, 76, 77) suggested that the first-dose vaccination with BNT162b2 mRNA, ChAdOx1, and Combined studies had reduced the COVID-19-related mortality rate by 68% (HR = 0.32, 95% CI: 0.23–0.45). However, people who were vaccinated with the first dose of BNT162b2 and ChAdOx1 showed 58% (HR = 0.42, 95% CI: 0.30–0.59) and 61% (HR = 0.39, 95% CI: 0.23–0.68) reduction in the mortality risk. Besides, the combined studies reduced the risk of COVID-19-related mortality by 91% (HR = 0.09, 95% CI: 0.01–0.64). The results of sub-group analysis for the first dose suggested that there was no difference between the effectiveness of different types of vaccines against COVID-19-related mortality rates (*p*−*value*_*subgroup*_ = 0.31) (Figure 6).

**Figure 7:**
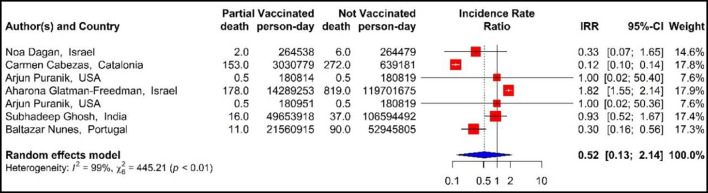
Effectiveness of vaccines against COVID-19-related mortality using incidence rate ratio in partial vaccinated individuals.

The results of examining the effectiveness of the first dose of vaccines against COVID-19-related mortality using IRR (33, 40, 42, 43, 48, 71), showed that the mortality rate in the people vaccinated with the first dose of BNT162b2 mRNA, mRNA-1273, ChAdOx1, and combined studies were reduced by 48% (IRR = 0.52, 95% CI: 0.13–2.14) (Figure 7).

#### Effectiveness of vaccines against SARS-CoV-2 infection, hospitalization, and mortality related to the COVID-19 in fully vaccinated individuals

##### SARS-CoV-2 infection

**Figure 8:**
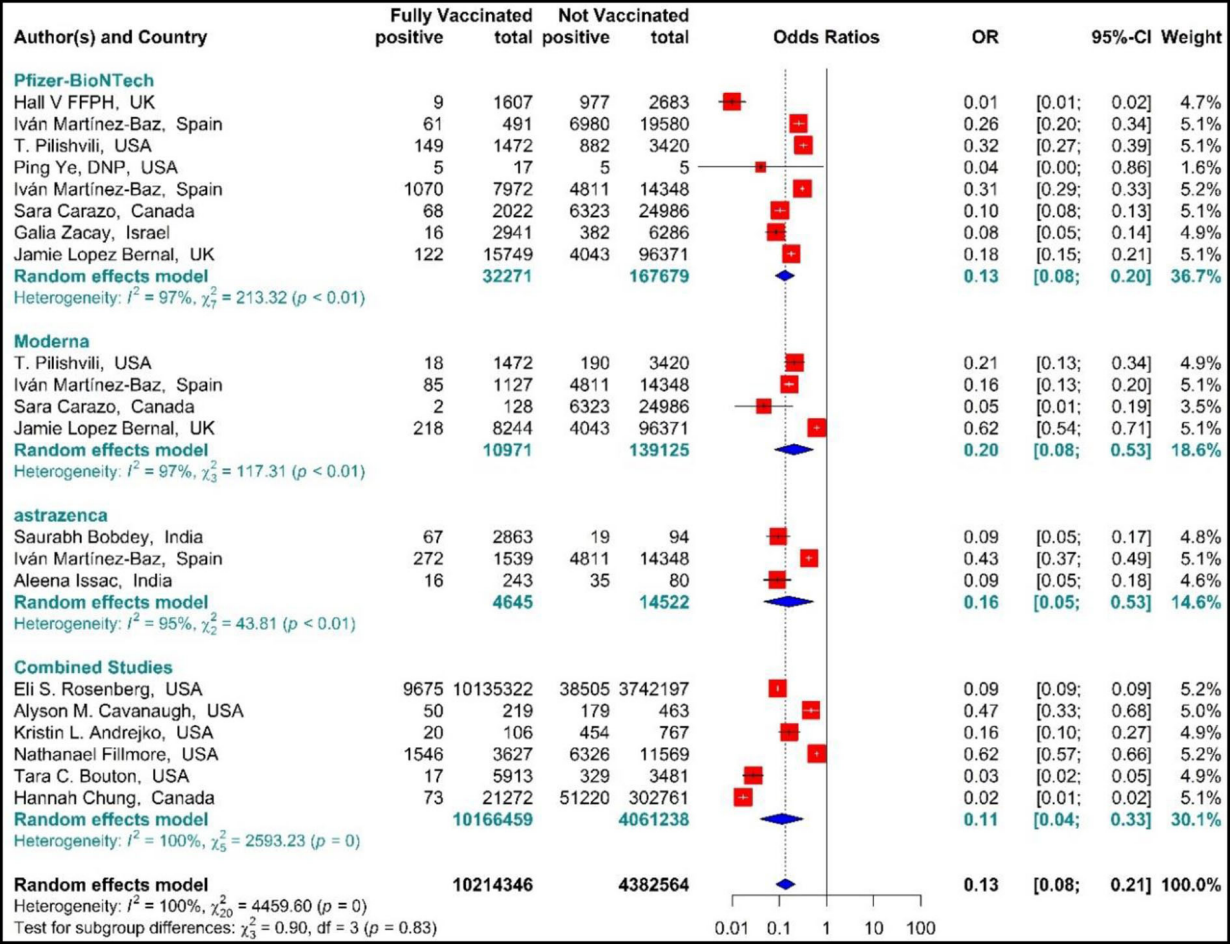
Effectiveness of vaccines against SARS-CoV-2 infection using odds ratio in Full vaccinated individuals.

The results of studies (34, 44, 51–54, 56, 57, 60–63, 67–70) are presented as forest plots using effect measure pooled OR in Figure 8. The results showed that the total effectiveness of the second dose of the vaccines (Fully vaccinated) against COVID-19 infection was 87% (OR = 0.13, 95% CI: 0.08–0.21); that is, the effectiveness of the second dose of BNT162b2 mRNA vaccine against COVID-19 infection was 87% (OR = 0.13, 95% CI: 0.08–0.20), that of mRNA vaccine-1273 was 80% (OR = 0.20, 95% CI: 0.08–0.53), and the effectiveness of the second dose of ChAdOx1 vaccine was 84% (OR = 0.16, 95% CI: 0.05–0.53). In addition, the combined studies that examined BNT162b2 mRNA, mRNA-1273, and ChAdOx1 vaccines reported an approximate effectiveness of 89% for the second doses (OR = 0.11, 95% CI: 0.04–0.33). The results of the sub-group analysis in the second dose suggested that there was no significant difference between different types of vaccines in terms of their effectiveness (*p*−*value*_*subgroup*_ = 0.83) (Figure 8).

**Figure 9:**
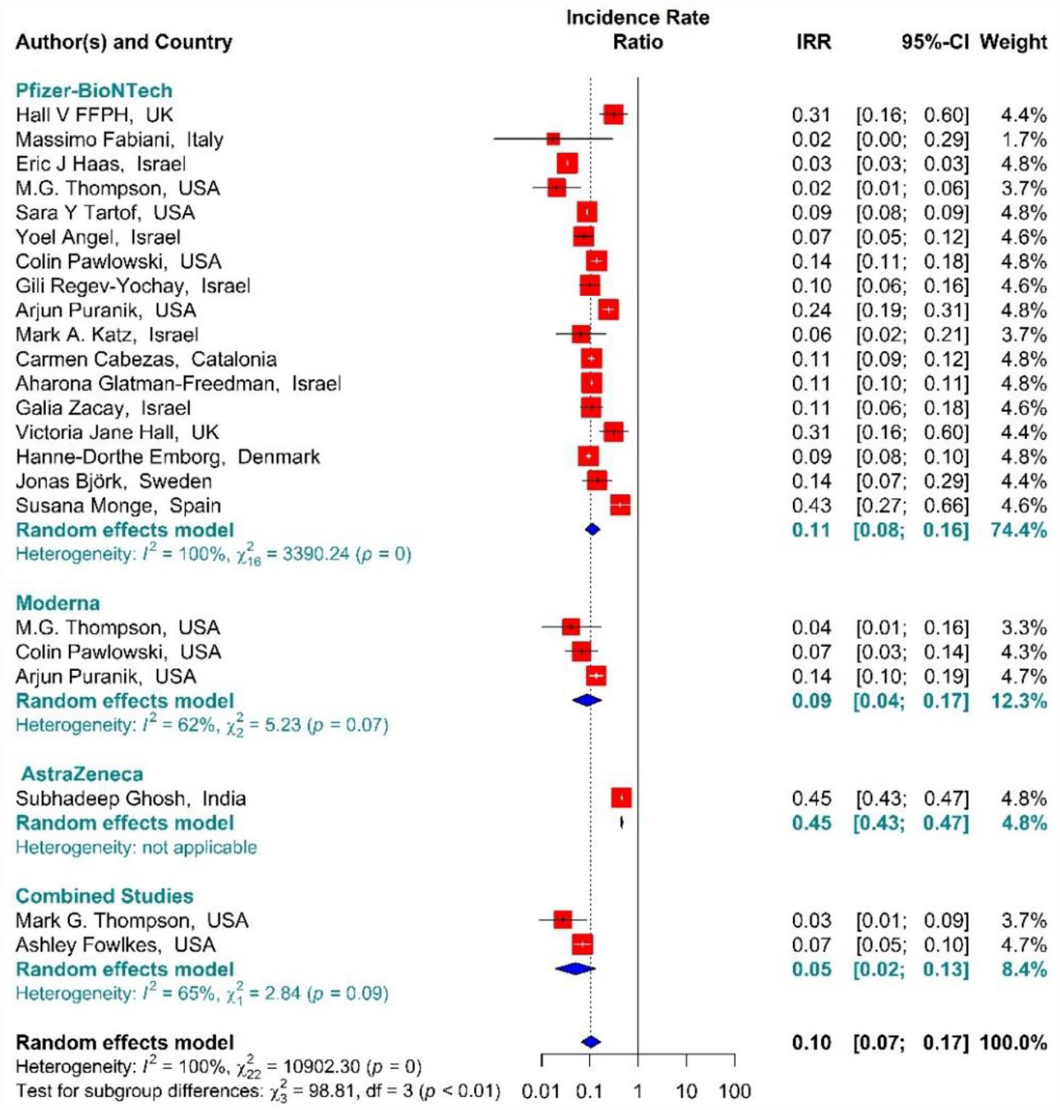
Effectiveness of vaccines against SARS-CoV-2 infection using incidence rate ratio in full vaccinated individuals.

In the people vaccinated with the second dose (fully vaccinated) of BNT162b2 mRNA, mRNA-1273, ChAdOx1, and combined studies, the rate of SARS-CoV-2 infection using IRR were reduced by 90% (IRR = 0.10, 95% CI: 0.07–0.17) (Figure 9) (11, 18, 19, 34, 35, 38, 39, 42–50, 80, 82). The reduction in SARS-CoV-2 infection rate in the individuals vaccinated with the second dose of BNT162b2 mRNA, mRNA-1273, and ChAdOx1 was 89% (IRR = 0.11, 95% CI: 0.08–0.16), 91% (IRR = 0.09, 95% CI: 0.04–0.17), and 55% (IRR = 0.45, 95% CI: 0.43–0.47), respectively (Figure 9). In the combined studies, the SARS-CoV-2 infection rate after the second dose had reduced by 95% (IRR = 0.05, 95% CI: 0.02–0.13). The results of the sub-group analysis in the second dose suggested that there was a difference between the effectiveness of different types of vaccines against SARS-CoV-2 infection (*p*−*value*_*subgroup*_ < 0.01) (Figure 9).

**Figure 10:**
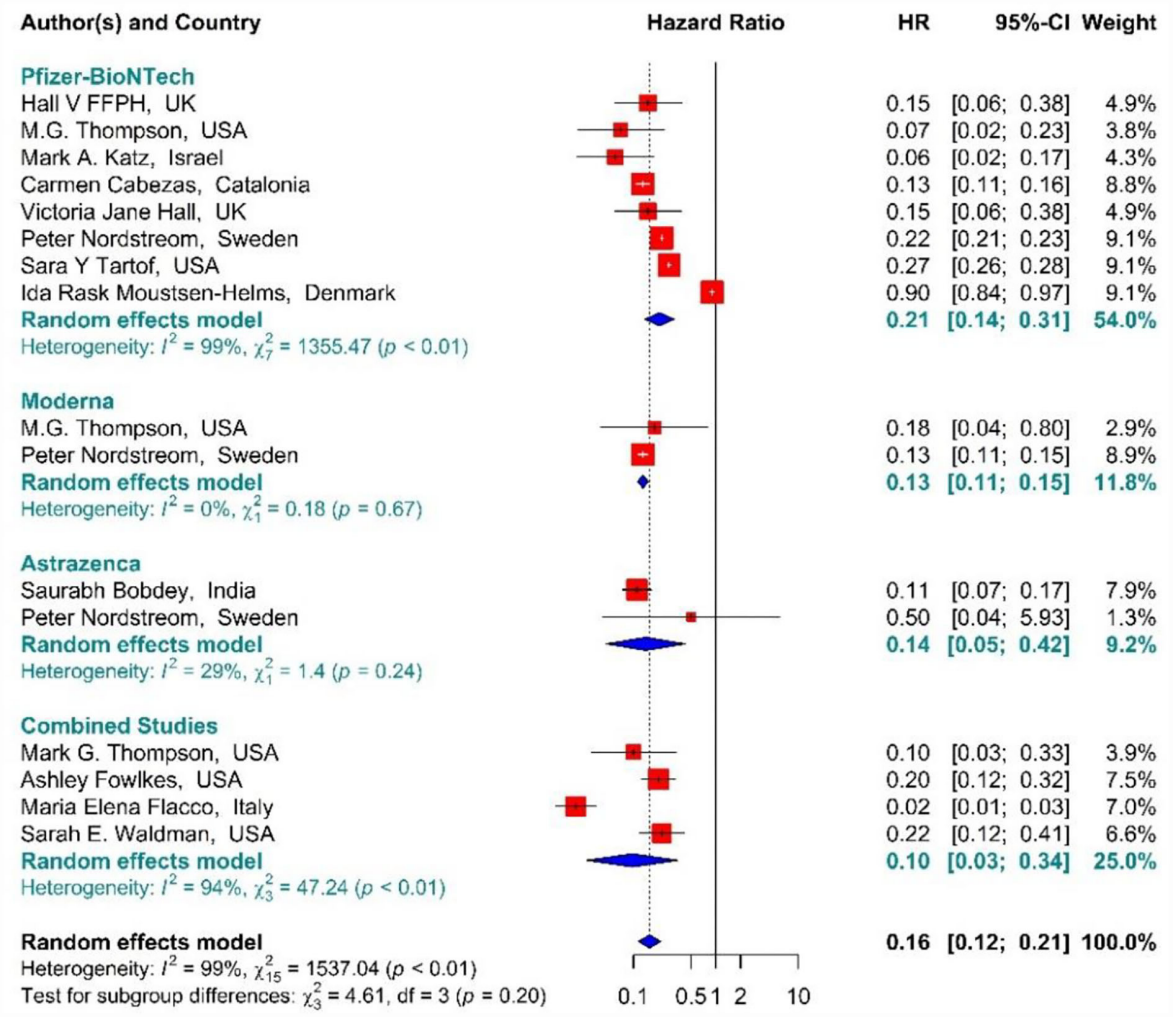
Effectiveness of vaccines against SARS-CoV-2 infection using hazard ratio in full vaccinated individuals.

In the individuals vaccinated with the second dose (Full vaccinated) of BNT162b2 mRNA, mRNA-1273, and ChAdOx1 vaccines, as well as the combined studies, the risk of SARS-CoV-2 infection using Hazard Ratio was reduced by 84% (HR = 0.16, 95% CI: 0.12–0.21) (Figure 10) (19, 34, 36, 41, 42, 49, 50, 61, 64, 79–81). However, the second dose of BNT162b2 mRNA, mRNA-1273, and ChAdOx1 vaccines reduced the risk of infection by 79% (HR = 0.21, 95% CI: 0.14–0.31), 87% (HR = 0.13, 95% CI: 0.11–0.15), and 86% (HR = 0.14, 95% CI: 0.05–0.42) respectively. Furthermore, the combined studies suggested that vaccination reduced the risk of SARS-CoV-2 infection in the individuals vaccinated with a second dose by 90% (HR = 0.10, 95% CI: 0.03–0.34). The results of the sub-group analysis in the second dose suggested that there was no difference between the effectiveness of different types of vaccines (*p*−*value*_*subgroup*_ = 0.2) (Figure 10).

##### Hospitalization

**Figure 11:**
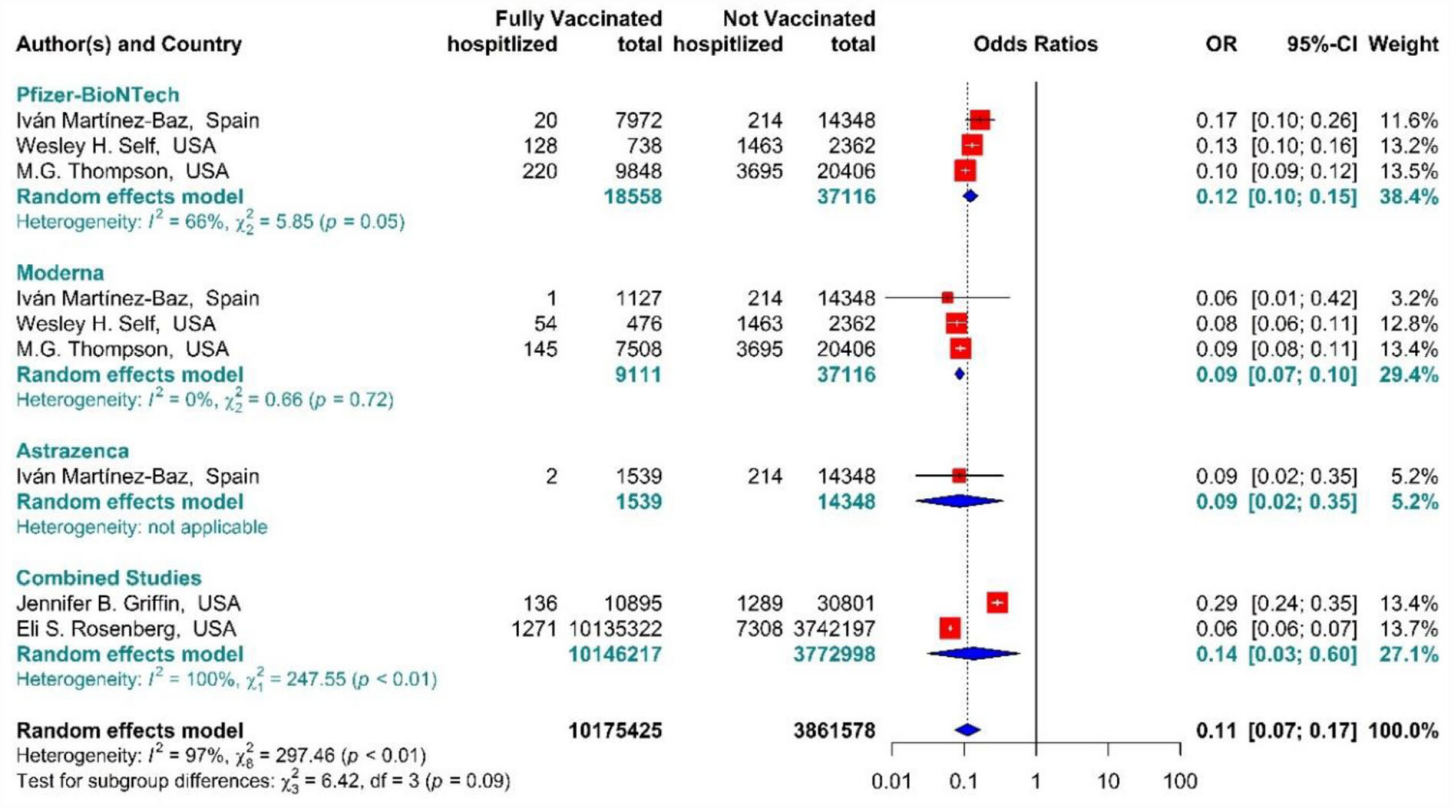
Effectiveness of vaccines against COVID-19-related hospitalization in full vaccinated individuals.

The total effectiveness of BNT162b2 mRNA, mRNA-1273, and ChAdOx1 vaccines, as well as the combined studies, for the second dose against COVID-19-related hospitalization was 89% (OR = 0.11, 95% CI: 0.07–0.17), while BNT162b2 mRNA, MRNA-1273, and ChAdOx1 vaccines had the effectiveness of 88% (OR = 0.12, 95% CI: 0.10–0.15), 91% (OR = 0.09, 95% CI: 0.07–0.10), and 91% (OR = 0.09, 95% CI: 0.02–0.35), respectively (Figure 11) (20, 56, 63, 72, 73). In addition, the effectiveness of the vaccines in the combined studies was 86% (OR = 0.14, 95% CI: 0.03–0.60). The results of the sub-group analysis in the second dose suggested that there was no significant difference between the effectiveness of different types of vaccines against hospitalization (*p*−*value*_*subgroup*_ = 0.09).

##### Mortality

**Figure 12:**
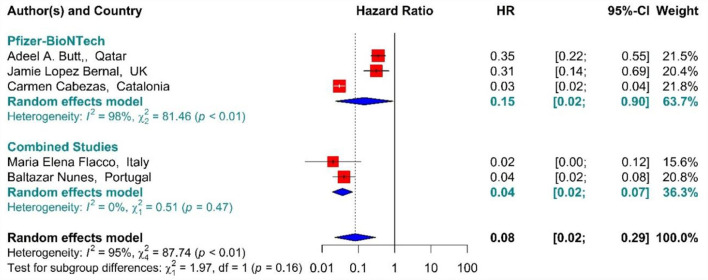
Effectiveness of vaccines against COVID-19-related mortality using hazard ratio in full vaccinated individuals.

In the individuals fully vaccinated with BNT162b2 mRNA as well as combined studies, the COVID-19-associated mortality risk using Hazard Ratio was reduced by 92% (HR = 0.08, 95% CI: 0.02–0.29) (Figure 12) (42, 64, 71, 75, 77). However, BNT162b2 mRNA vaccine and the combined studies reduced the risk by 85% (HR = 0.15, 95% CI: 0.02–0.90) and 96% (HR = 0.04, 95% CI: 0.02–0.07), respectively. The results of the sub-group analysis in the second dose showed that there was no difference between the effectiveness of different vaccines against COVID-19-related death (*p*−*value*_*subgroup*_ = 0.16) (Figure 12).

**Figure 13:**
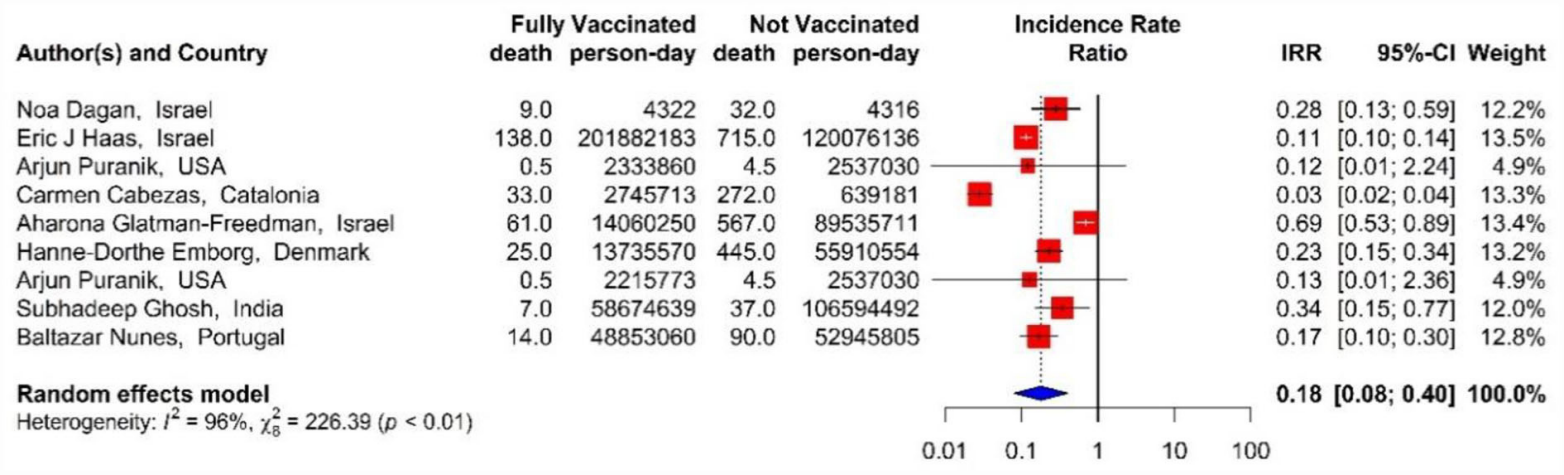
Effectiveness of vaccines against COVID-19-related mortality using incidence rate ratio in full vaccinated individuals.

In addition, the effectiveness of BNT162b2 mRNA, mRNA-1273, and ChAdOx1 vaccines, as well as the combined studies, against COVID-19-related mortality using IRR in the second dose was 82% (IRR = 0.18, 95% CI: 0.08–0.40) (Figure 13) (18, 33, 40, 42, 43, 45, 48, 71).

#### Sub-group analysis by study design

The results of the sub-group analysis with regard to the type of studies suggested that there was no statistically significant difference between case-control studies, prospective studies, and retrospective studies in terms of the effectiveness of vaccines against SARS-CoV-2 infection, hospitalization rate, and mortality associated with COVID-19 (Supplementary File 1 in Figures 1–12).

#### Quality assessment, sensitivity analysis, and publication bias

Supplementary File 1 in Tables 1, 2 shows the quality of the included articles according to *NOS* (due to limited space and word counting, the results of the NOS tool are provided as Supplementary File 2). The results of the sensitivity analysis showed that there was no significant difference between the studies included in the meta-analysis (Supplementary File 2 in Figures 1–12). In addition, publication bias in the studies included in the meta-analysis was investigated through Funnel Plot and Eggers' test, the results of which showed no publication bias in the studies included in the meta-analysis (Eggers' test *P*-value > 0.05) (Supplementary File 2 in Figures 13–15).

## Discussion

In the present meta-analysis of the observational studies, we aimed to evaluate the effectiveness of vaccination in reducing the incidence of SARS-CoV-2 infection as well as mortality and hospitalization.

Although some systematic reviews and meta-analyses of RCT studies have been conducted in the field of vaccination and COVID-19, none of them has wholly and comprehensively investigated the effective role of vaccination for COVID-19 on the incidence, hospitalization, and mortality of patients. On the other hand, focusing on the influential role of injectable doses of vaccine in observational studies was fully investigated in this meta-analysis, which was not comprehensively examined in the previous studies.

The results supported the findings of phase 3 of the clinical trials on the effectiveness of BNT162b2 mRNA, mRNA-1273, and ChAdOx1 vaccines (12, 83, 84). More precisely, previously, the effectiveness of the first and second doses of BNT162b2 mRNA vaccine against SARS-CoV-2 infection was reported to be 82% and 95%, respectively (12), and we found that the pooled estimates of the effectiveness against SARS-CoV-2 were 72 and 89%, respectively. Also, the effectiveness of ChAdOx1 and mRNA-1273 vaccines against the incidence of infection was estimated at about 51 and 69% in the first dose and 84% and 80% in the second dose, respectively. These results are consistent with the previous studies (33, 83, 84).

Notably, the observed difference in the effectiveness of the first and the second doses could be due to the fact that those corona vaccines that were designed as two-dose regimens are suggested to be injected at regular intervals to achieve the highest immunity. Several studies suggested that receiving only one dose of the vaccine creates a partial immunity response and might provide a shorter period of immunity than receiving full doses (18, 34, 78, 85, 86).

As such, the pooled increased effectiveness of the studied vaccines against SARS-CoV-2 infection after the second dose was 16% (from 71% in the first dose to 87% after the second dose). The increased effectiveness of the BNT162b2 mRNA vaccine in the second dose compared to the first one was 15%, and that of mRNA-1273 and ChAdOx1 vaccines was 11% and 33%, respectively. Also, the difference between the effectiveness of the two doses of vaccines against the incidence of SARS-CoV-2 infection in the studies that examined the vaccines heterogeneously (a combination of COVID-19 vaccines on the general population) was 11%.

Interestingly, especially after the second dose, the effectiveness of the vaccines increased significantly with the increased post-vaccination follow-up periods. Accordingly, Hunter and Brainard (87) reported relatively high effectiveness of the first dose of BNT162b2 mRNA 21 days after the second injection. The Hunter's study results indicated that high effectiveness of the second dose of COVID-19 vaccines against COVID-19 infection, hospitalization, and mortality was achieved between 20 and 30 days after the first dose.

Although the present study aimed at evaluating the effectiveness of homologous vaccines, there were some studies that examined the effectiveness of different combinations of vaccines in different populations. For example, few studies evaluated the immunity of populations that were vaccinated with BNT162b2 mRNA, mRNA-1273, and ChAdOx1 (and even Ad26.COV2.S in some rare cases), the results of which showed significant improvement in the effectiveness of the vaccines. In a study of combined vaccines, Nordstrom et al. (80) showed that vaccines' effectiveness varied from 67 to 79% depending on the types administered. The results of our meta-analysis on the effectiveness of combined vaccines were also consistent with the study by Nordstrom et al. and strengthened the hypothesis of the better effect of combined vaccines against SARS-CoV-2 infection.

Considering different variants of COVID-19, although the effectiveness of COVID-19 vaccines against *alpha* and *delta* variants is reported to be lower, the effectiveness of full vaccination against these variants has been revealed to be acceptably high (60). In an observational study, Haas et al. (18) reported the high effectiveness of two doses of BNT162b2 mRNA vaccine against the *B.1.1.7* variant of SARS-CoV-2 infection, hospitalization, and mortality. However, another study on the effects of COVID-19 vaccines on *delta* variants did not observe a significant effect 28 days after the first dose (88). Our meta-analysis also suggested that the effect of complete vaccination on the reduction of the incidence of infection, hospitalization, and mortality is high regardless of SARS-CoV-2 variants (88). Moreover, the effectiveness of complete COVID-19 vaccination in reducing the rate of hospitalization in our study confirmed the results of the previous studies on the prevention of COVID-19-related hospitalization (18, 20, 59). The biggest difference in the effectiveness of the two vaccine doses against hospitalization was related to BNT162b2 mRNA and mRNA-1273 with 35 and 29% increase, respectively, in the effectiveness of vaccines after the second doses. Also, administering the second dose injection was associated with 21% decrease in the risk of COVID-19 mortality compared to the first dose (68% in the first vs. 89% in the second doses).

It is suggested that the effectiveness of vaccines in the community is an ecological issue, and separating it from non-medical measures such as quarantine and wearing masks is difficult. However, various studies reported high levels of vaccine effectiveness even after the reopening of communities (18). The other concern in evaluating the study's results is the test policies for vaccinated and unvaccinated individuals, which vary from community to community. For example, in Israel, SARS-CoV-2 testing policy was different for unvaccinated and vaccinated individuals; the vaccinated individuals must provide evidence of being in contact with PCR-positive persons or returning from abroad (33). This may lead to an overestimation of vaccine effectiveness. Moreover, vaccinated and unvaccinated people have different behaviors in seeking healthcare and taking diagnostic tests for COVID-19, which can, in turn, affect the effectiveness of the vaccine. People who have refused to be vaccinated are also less likely to take a diagnostic test, which can lead to underestimated vaccine effectiveness. Other reasons that can affect the validity of the results is different follow-up times in various studies, the interval between the first and the second doses of vaccines, and the fact that the persons may delay taking the second dose of vaccine deliberately or due to a lack of logistic and technical preparations. This can in turn affect the vaccine's effectiveness (18, 89).

Although the differences were not significant, the results of the present study showed that the effectiveness of the vaccines varies in different studies. For example, several prospective cohort studies showed higher effectiveness compared to retrospective cohorts, and they both showed higher effectiveness than case-control studies. Although, it has been suggested that the best studies to evaluate the effectiveness of vaccines are randomized clinical trials, because they strongly differentiate the protective effect of vaccines at the individual level (90), non-randomized studies played a major role in estimating the effectiveness of vaccines during the pandemic. For example, a Scottish retrospective cohort study provided promising findings on the effectiveness of the first doses of Pfizer and AstraZeneca vaccines in Scotland (86). A considerable reason for the importance of non-randomized studies is that different variants cannot be randomly divided into different groups and thus, non-randomized studies are a good alternative to clinical trials to estimate the effectiveness of vaccines against new variants. In addition, negative test studies are considered as one of the most appropriate types of studies that properly reduce the disruptive effect of health seeking behavior in the compared groups (91), as a recent negative test study in Canada provided evidence of the effectiveness of Pfizer, Moderna, and AstraZeneca vaccines against alpha, beta, gamma, and delta variants (92).

This study has some strengths and limitations to be noted. Among the strengths of the present study is that we examined all aspects of the effectiveness of vaccination against the incidence of COVID-19, including SARS-CoV-2 infection, hospitalization, and mortality from COVID-19. Since the quality of meta-analyses is largely reliant on the quality of the original studies included, in our study, we included high-quality studies from different parts of the world with relatively large sample sizes and cohort studies with appropriate follow-ups resulting in increasing the validity of the results. The presence of studies from different regions may influence the generalizability of our study results. Notably, the important procedures such as searching studies, data extraction, and quality assessment were independently performed and reviewed by two experts in the field of secondary studies. Despite the significance of our findings about the effectiveness of COVID-19 vaccines in reducing the incidence of infection, hospitalization, and mortality associated with COVID-19, this study had a number of limitations, including the effects of different vaccines on different variants, the possibility of vaccination in a specific age group, or vaccine hesitancy, which refers to the delay in accepting or refusing available vaccination, which indicated that non-vaccinated people had a higher risk of SARS-CoV-2 infection and we had no access to such data. The confounding of the background factors may, however, have a limited influence on our results when using HR adjusted in the included trials. Another disadvantage is that the less investigated COVID-19 vaccines did not have the chance to be assessed and hence were not included in our analysis. As a result, further research is needed to validate the efficacy of vaccinations that have received less attention.

## Conclusion

The results of this meta-analysis indicated that vaccination against COVID-19 with BNT162b2 mRNA, mRNA-1273, and ChAdOx1, and also their combination, was associated with a favorable effectiveness against SARS-CoV-2 incidence rate, hospitalization, and mortality rate in the first and second doses in different populations. On the other hand, due to the higher effectiveness of the second dose of vaccines, compared to the first dose, in reducing the incidence rate of infection, mortality rate, and hospitalization associated with COVID-19, we suggest that to prevent the severe form of the disease in the future, and, in particular, in the coming epidemic picks, vaccination could be compulsory for high-risk individuals. We, also, strongly suggest more research on the durability of immunity after booster vaccines and the effect of booster doses on the effectiveness of COVID-19 vaccines on the incidence rate, mortality rate, and hospitalization rate of the disease. Also, more research on the effectiveness of booster doses with different vaccines on the new variants is highly recommended. Likewise, our results would apply to health policymakers and stakeholders to encourage people to accept the effects of vaccines and minimize vaccine hesitancy in the prevention of severe forms of the disease.

## Data availability statement

The original contributions presented in the study are included in the article/Supplementary material, further inquiries can be directed to the corresponding author/s.

## Author contributions

KR, RS, and MD contributed to the design and implementation of the study, analysis, and interpretation of data, and were involved in drafting the manuscript. HD and MK contributed to the assessing quality of studies. MFor and RO contributed to the interpretation of data and were involved in drafting and revising the manuscript. MS and MR contribute to the data extractions and data management. All authors read and approved the final manuscript.

## Conflict of interest

The authors declare that the research was conducted in the absence of any commercial or financial relationships that could be construed as a potential conflict of interest.

## Publisher's note

All claims expressed in this article are solely those of the authors and do not necessarily represent those of their affiliated organizations, or those of the publisher, the editors and the reviewers. Any product that may be evaluated in this article, or claim that may be made by its manufacturer, is not guaranteed or endorsed by the publisher.
