# Supplementary material for: The effectiveness of COVID-19 vaccines in reducing the incidence, hospitalization, and mortality from COVID-19: A systematic review and meta-analysis
Source: Front Public Health. 2022 Aug 26;10:873596. doi: 10.3389/fpubh.2022.873596 (PMC9459165; doi:10.3389/fpubh.2022.873596)
Supplement: Supplementary file 1 [file Data_Sheet_1.docx]

**Supplementary.1**
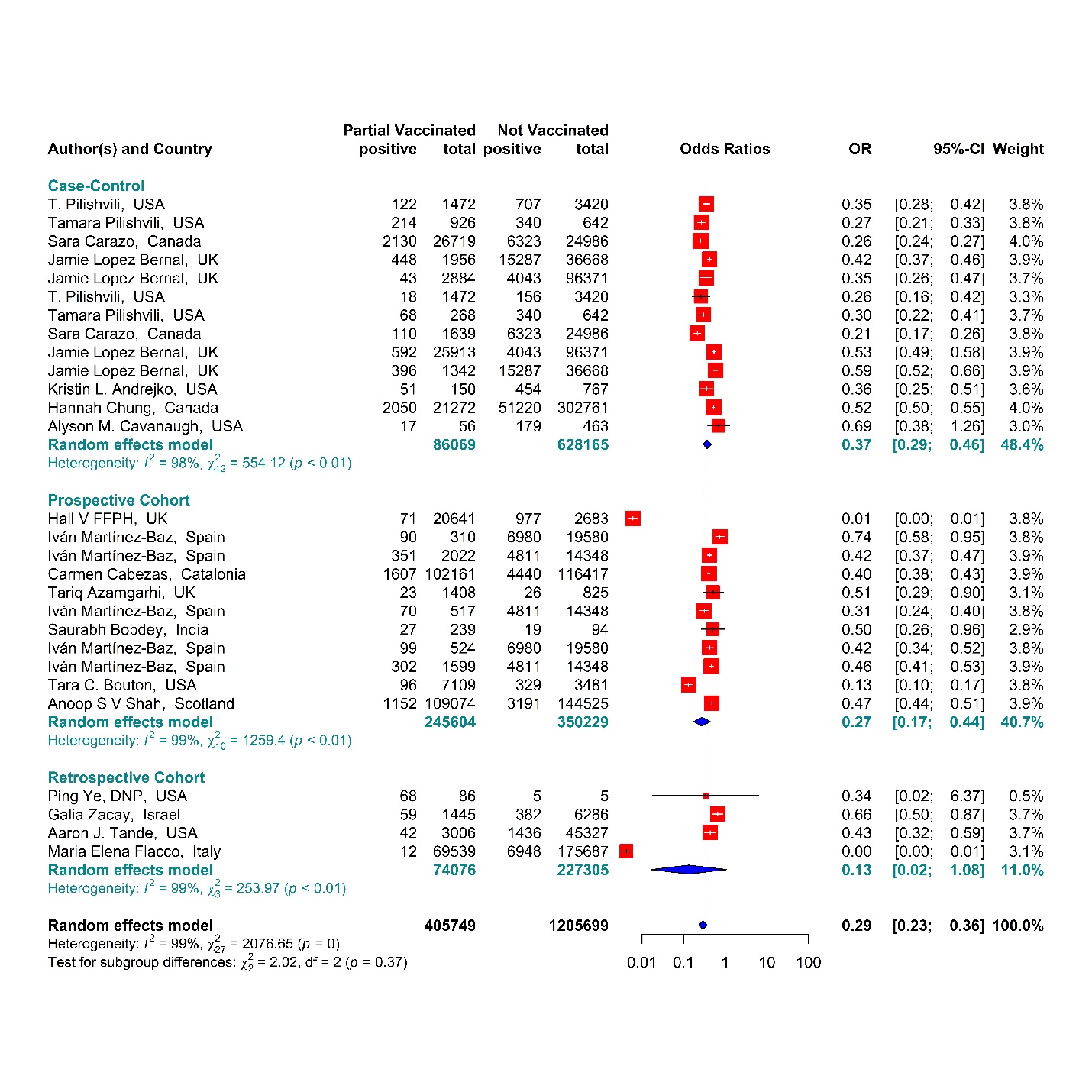


Figure 1. Partial vaccinated effectiveness of BNT162b2 mRNA, mRNA-1273, and ChAdOx1 vaccines against SARS-COV 2 infection by type of study


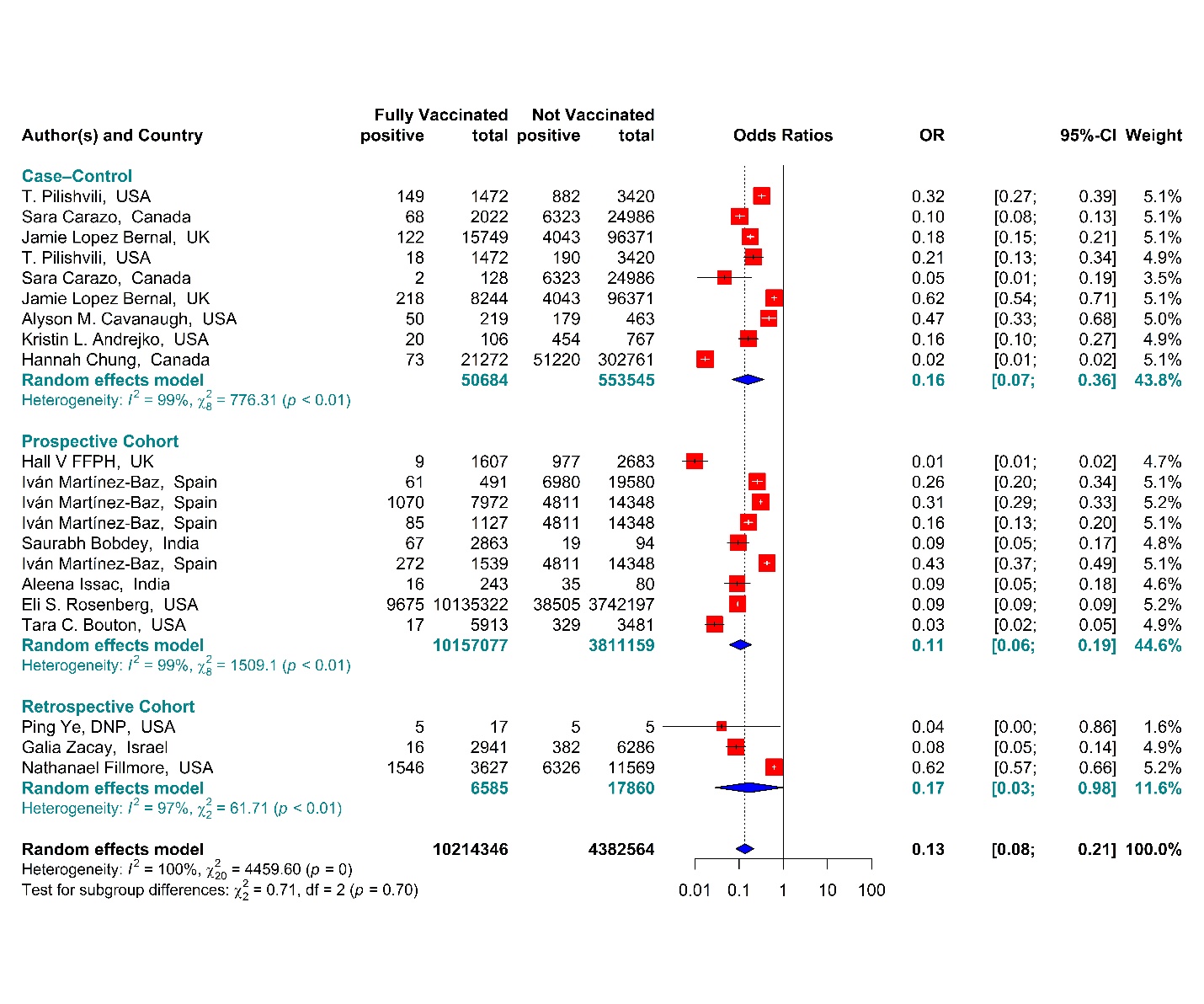


Figure 2. Full vaccinated effectiveness of BNT162b2 mRNA, mRNA-1273, and ChAdOx1 vaccines against SARS-COV 2 infection by type of study


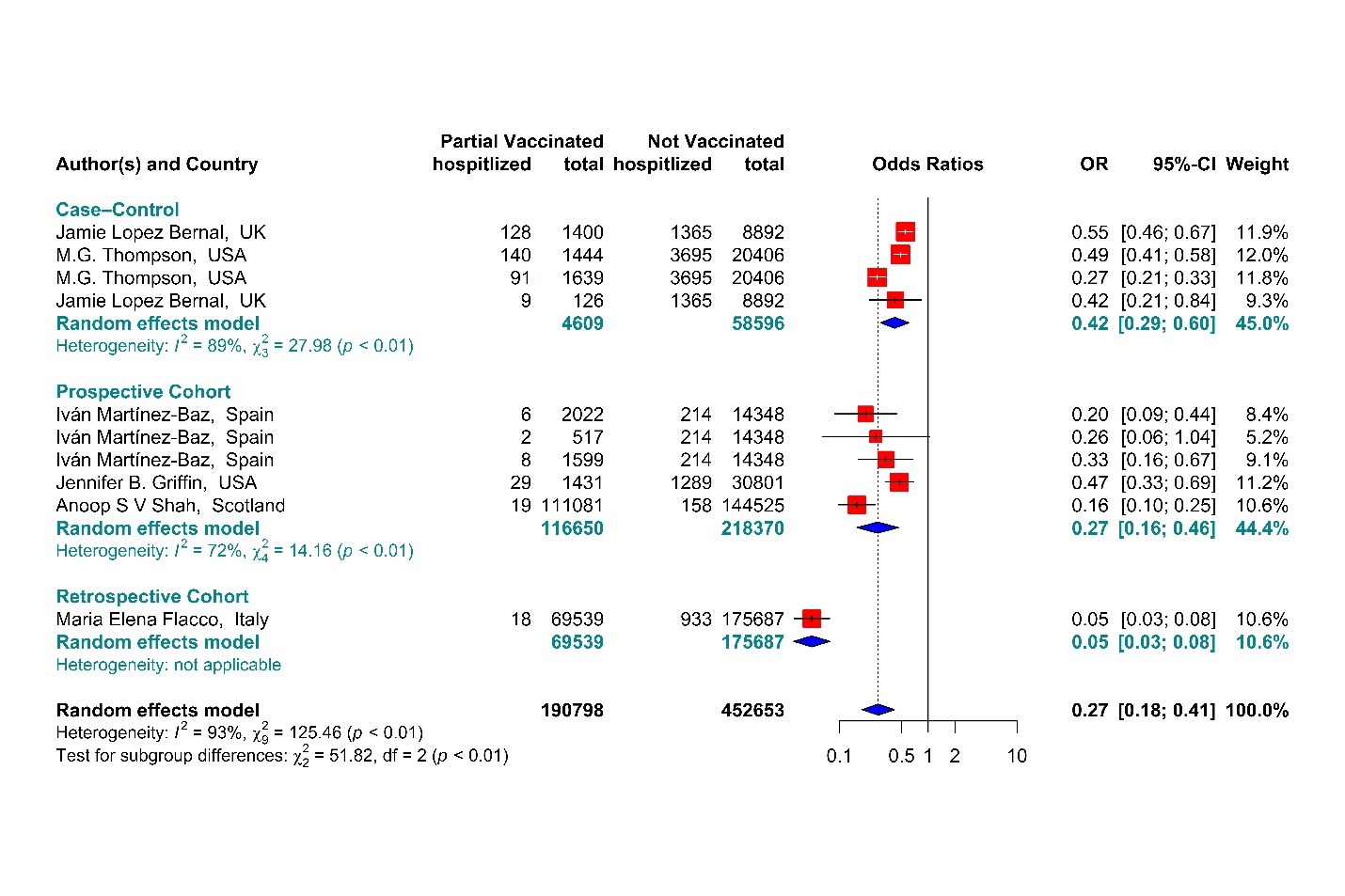


Figure 3. Partial effectiveness of BNT162b2 mRNA, mRNA-1273, and ChAdOx1 vaccines against COVID-19-related hospitalization by type of study


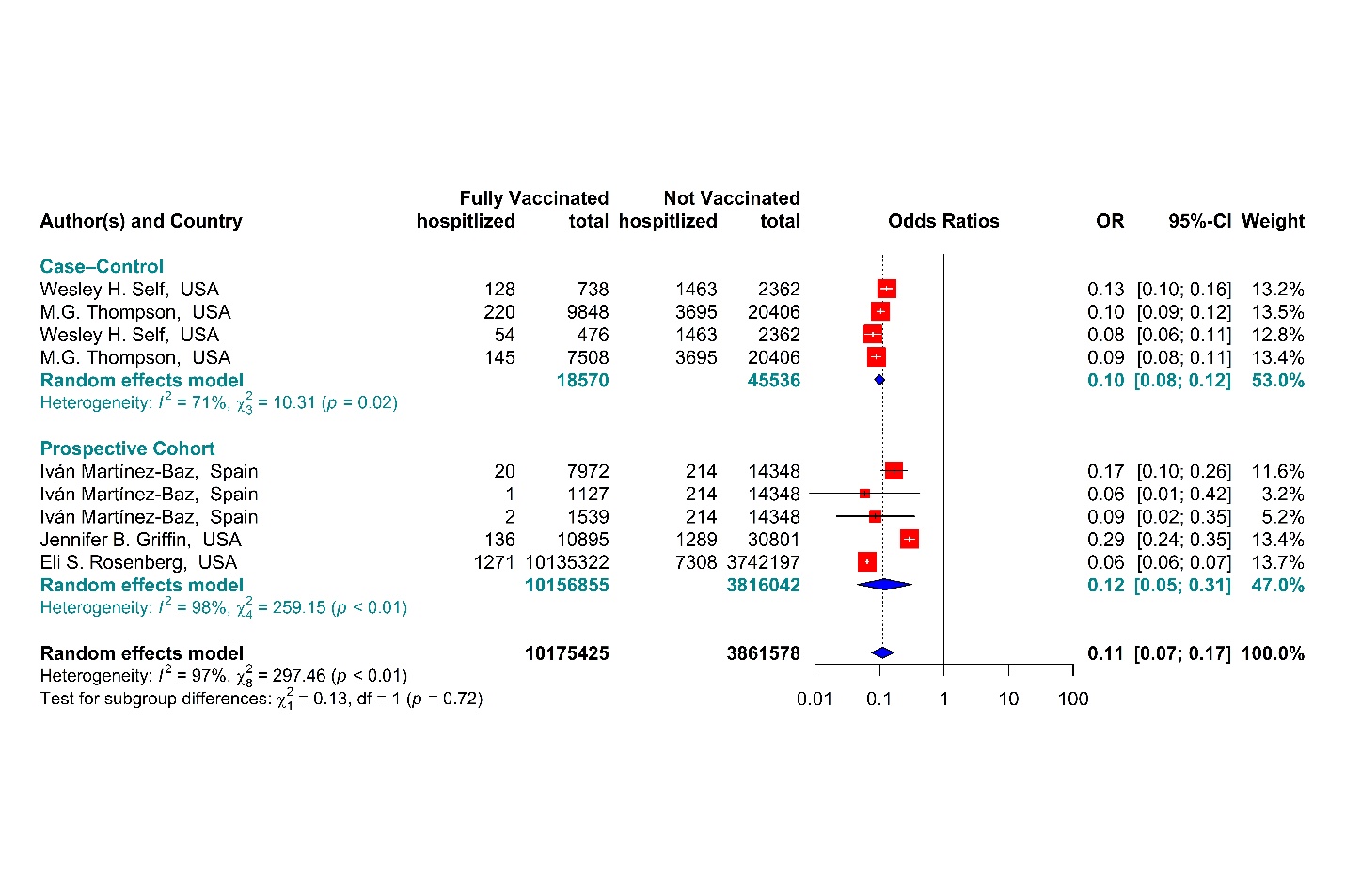


Figure 4. Full effectiveness of BNT162b2 mRNA, mRNA-1273, and ChAdOx1 vaccines against COVID-19-related hospitalization by type of study


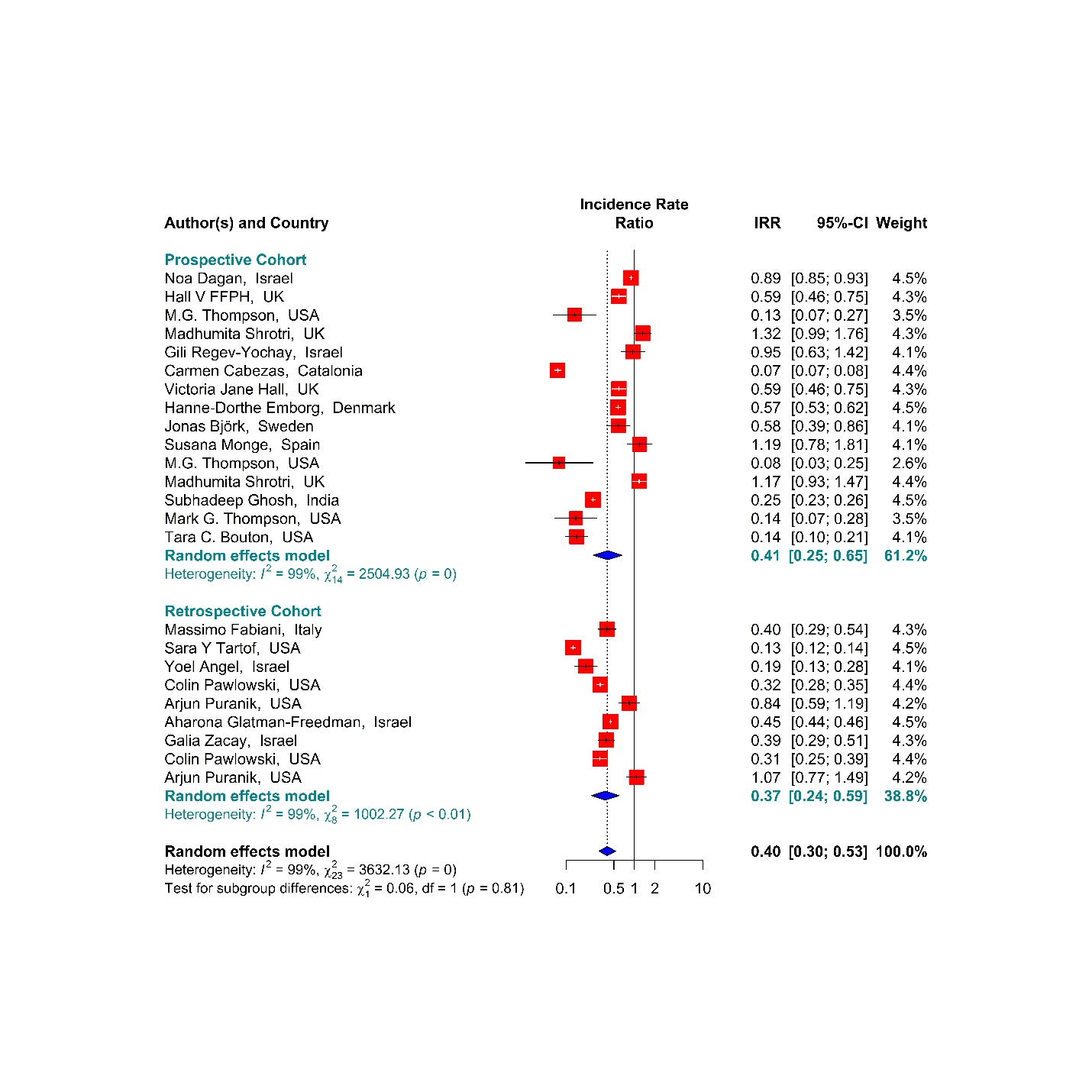


Figure 5. Effectiveness of vaccines against SARS-COV 2 infection using Incidence rate ratio in partial vaccinated individuals by type of study


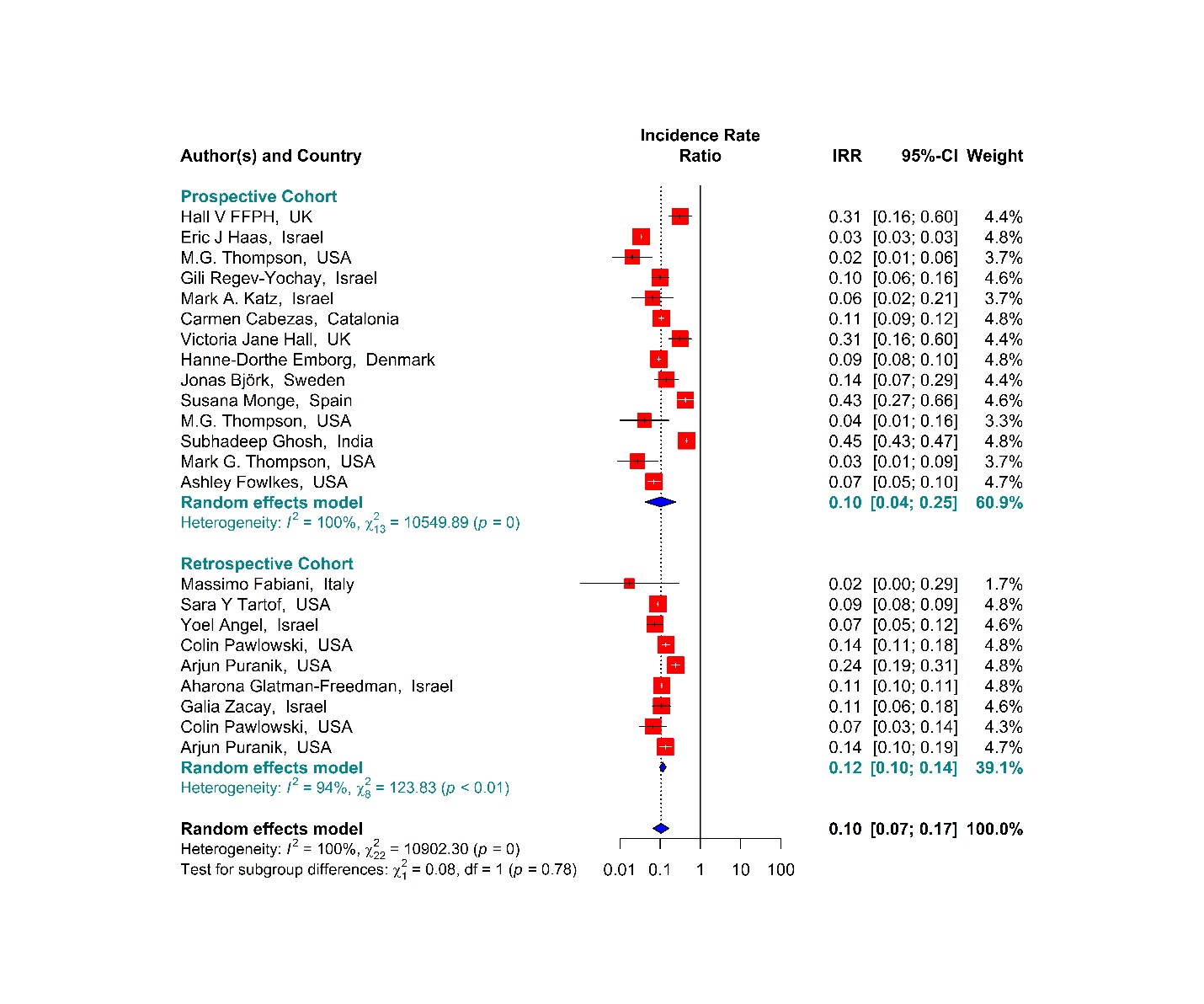


Figure 6. Effectiveness of vaccines against SARS-COV 2 infection using Incidence rate ratio in full vaccinated individuals by type of study


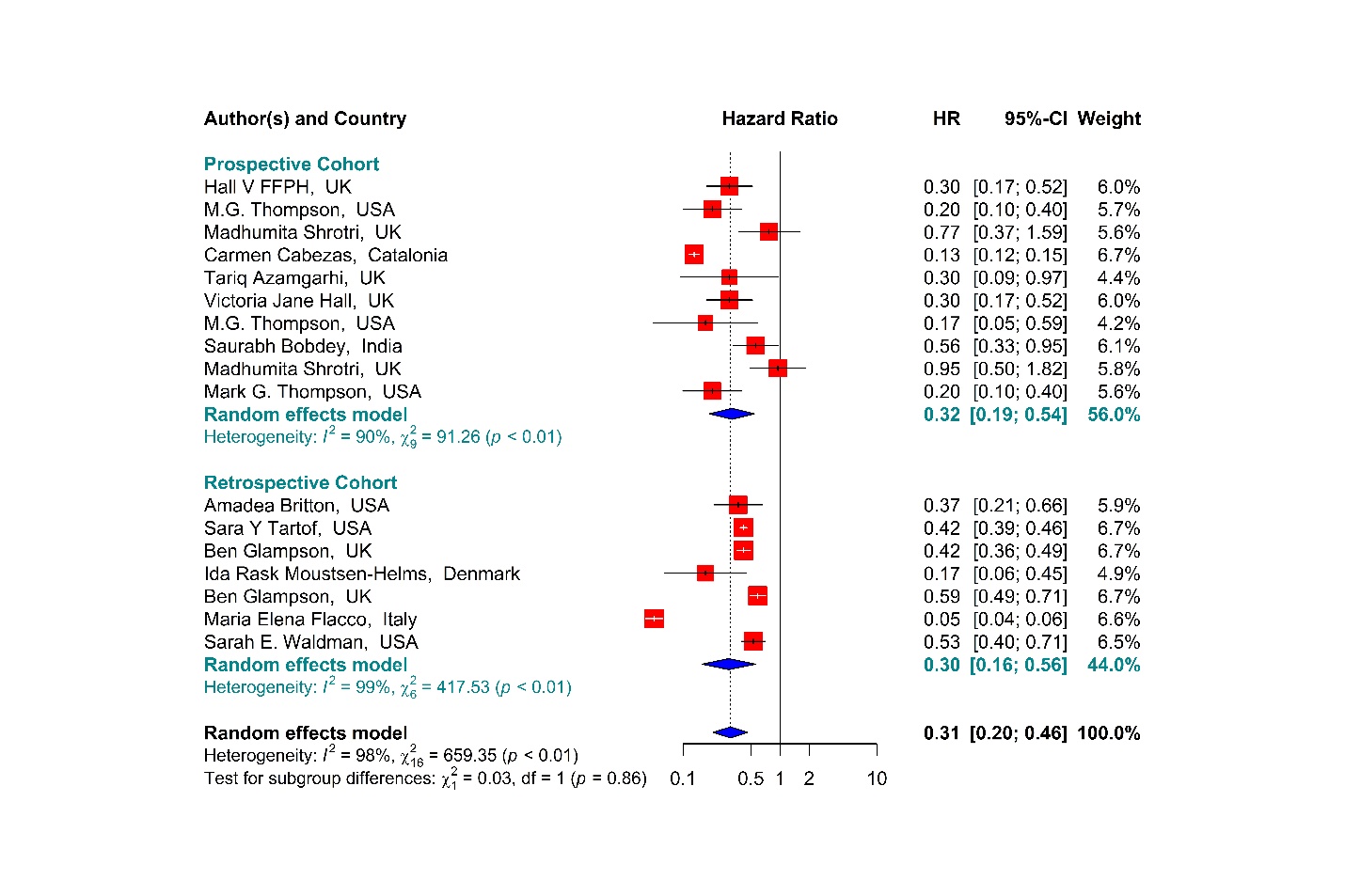


Figure 7. Effectiveness of vaccines against SARS-COV 2 infection using Hazard ratio in partial-vaccinated individuals by type of study


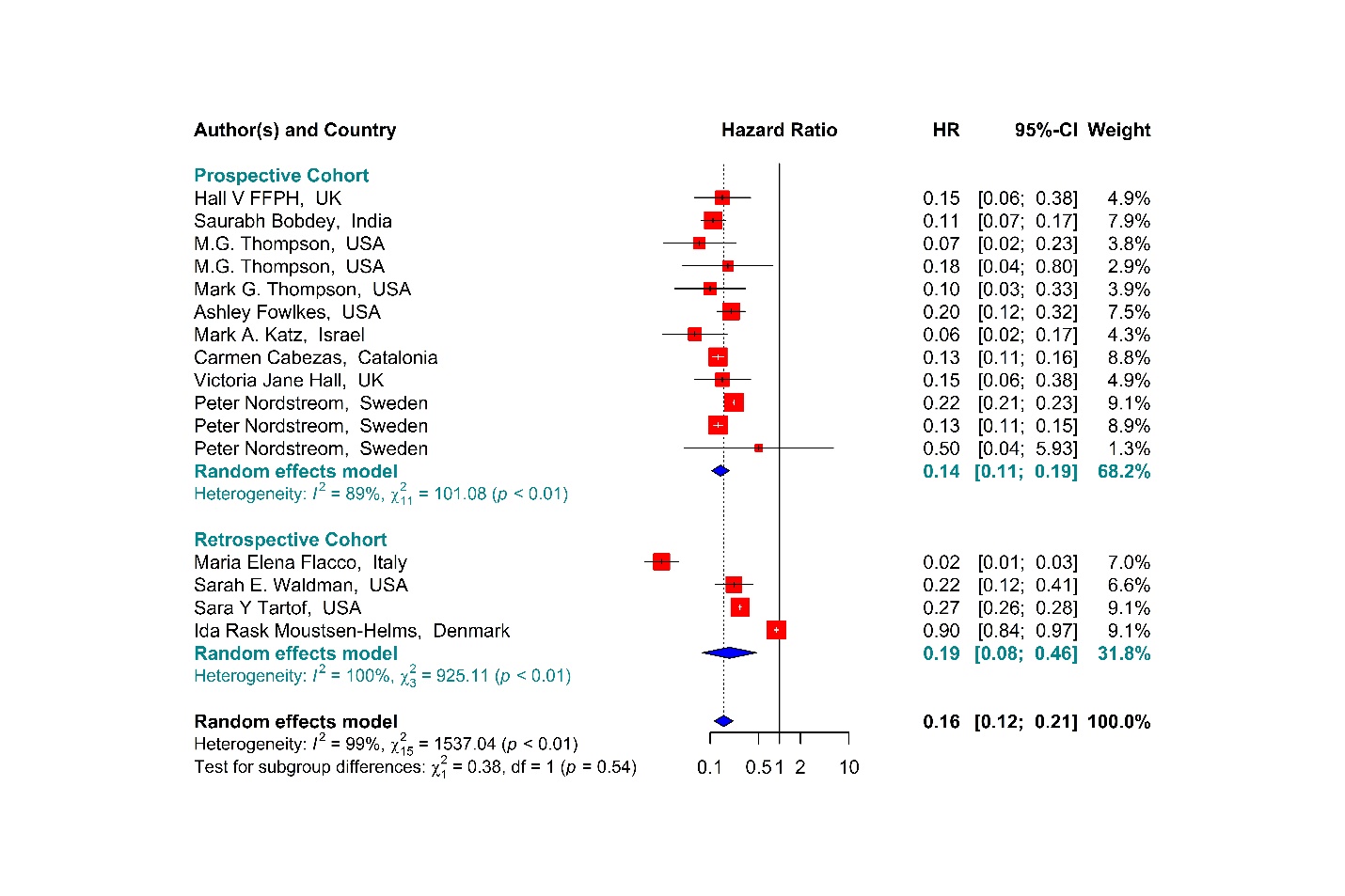


Figure 8. Effectiveness of vaccines against COVID-19 infection using Hazard ratio in full vaccinated individuals by type of study


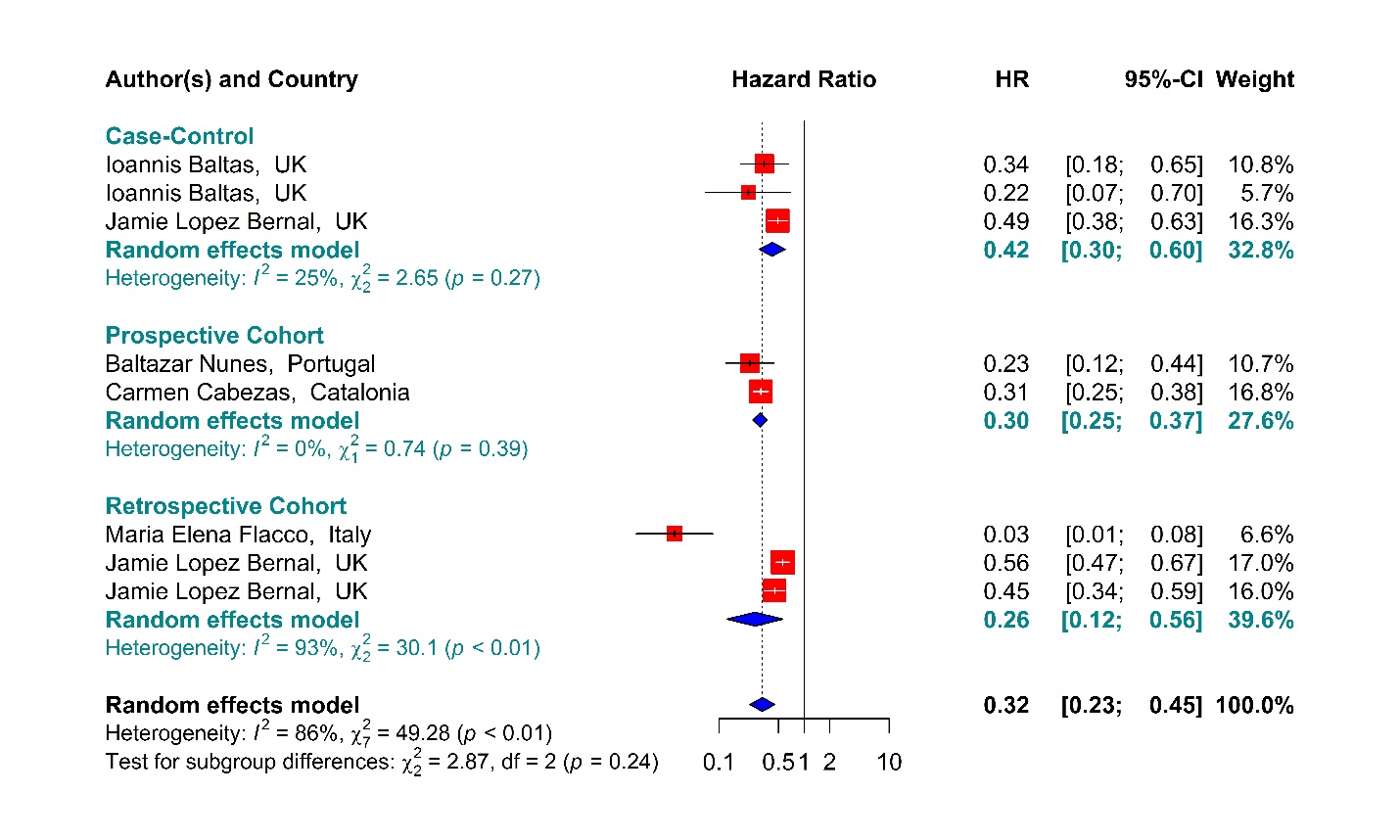


Figure 9. Effectiveness of vaccines against COVID-19-related mortality using Hazard ratio in partial-vaccinated individuals by type of study


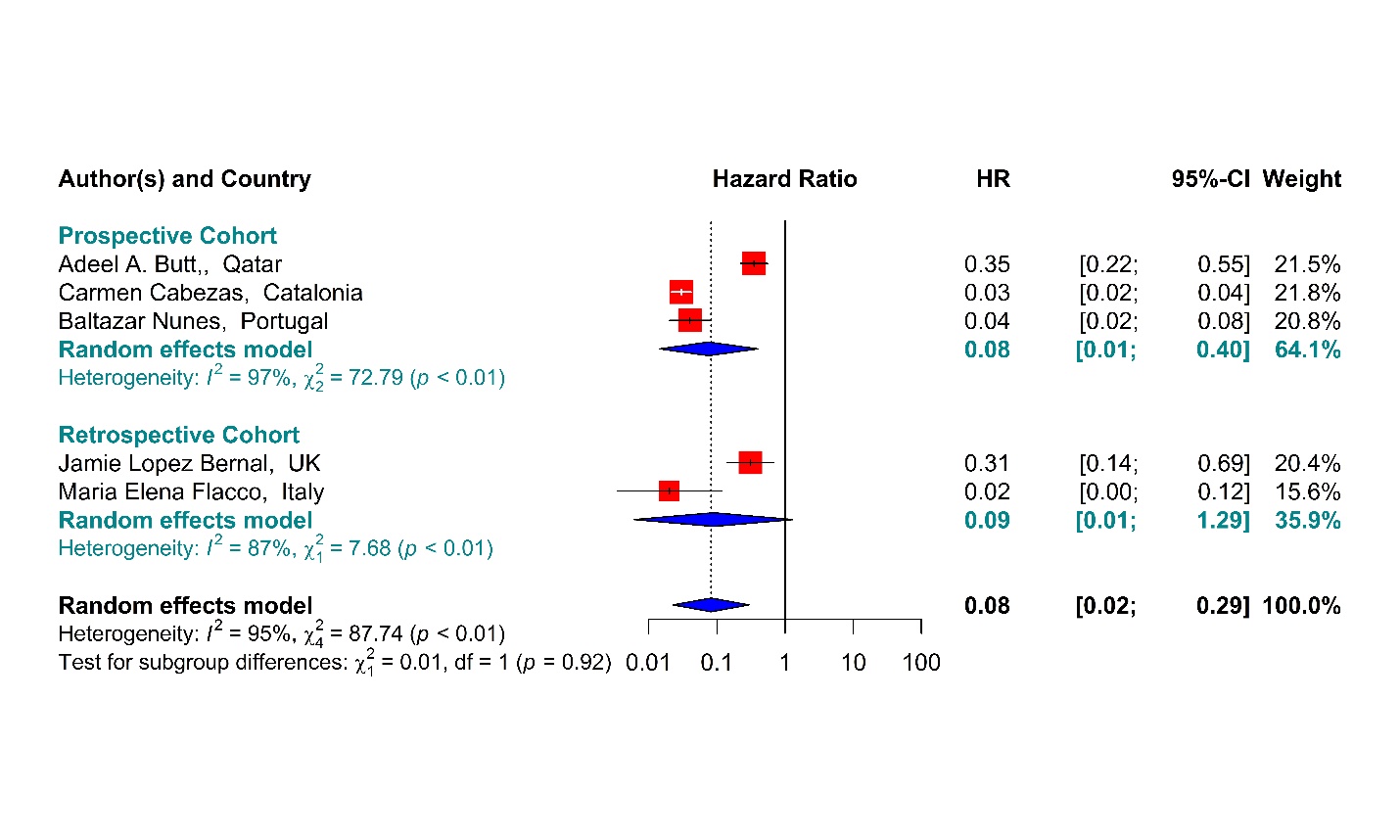


Figure 10. Effectiveness of vaccines against COVID-19-related mortality using Hazard ratio in full-vaccinated individuals by type of study


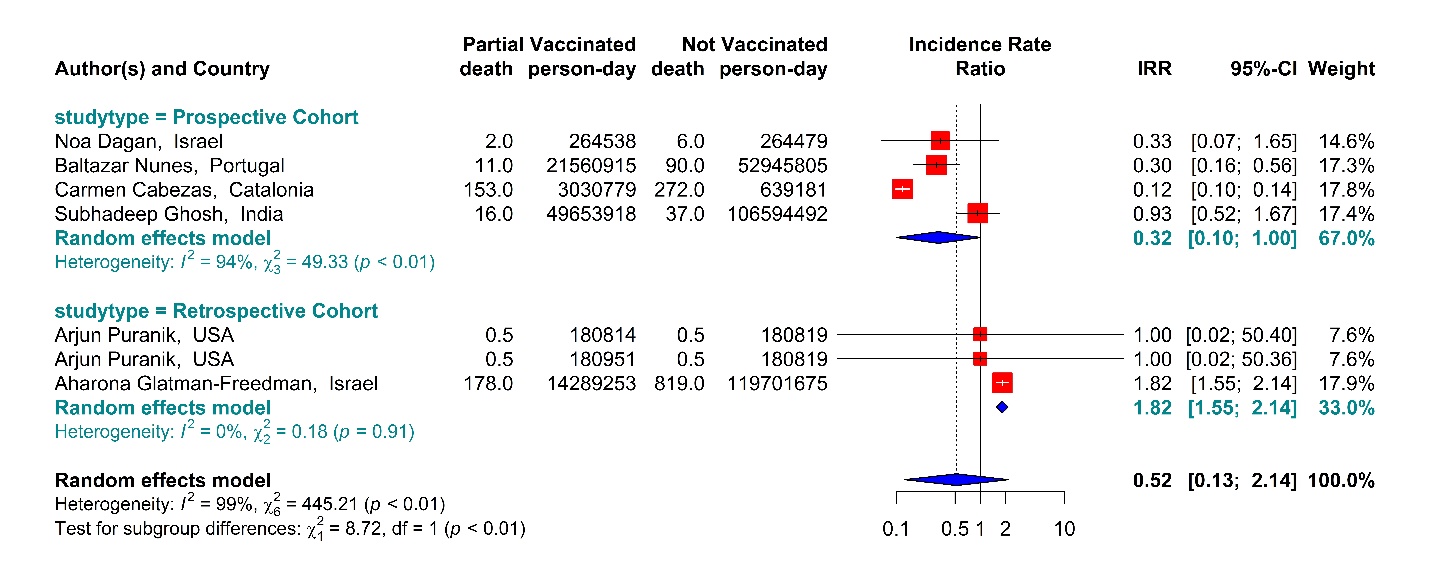


Figure 11. Partial effectiveness of vaccines against COVID-19-related mortality using Incidence Rate Ratio by type of study


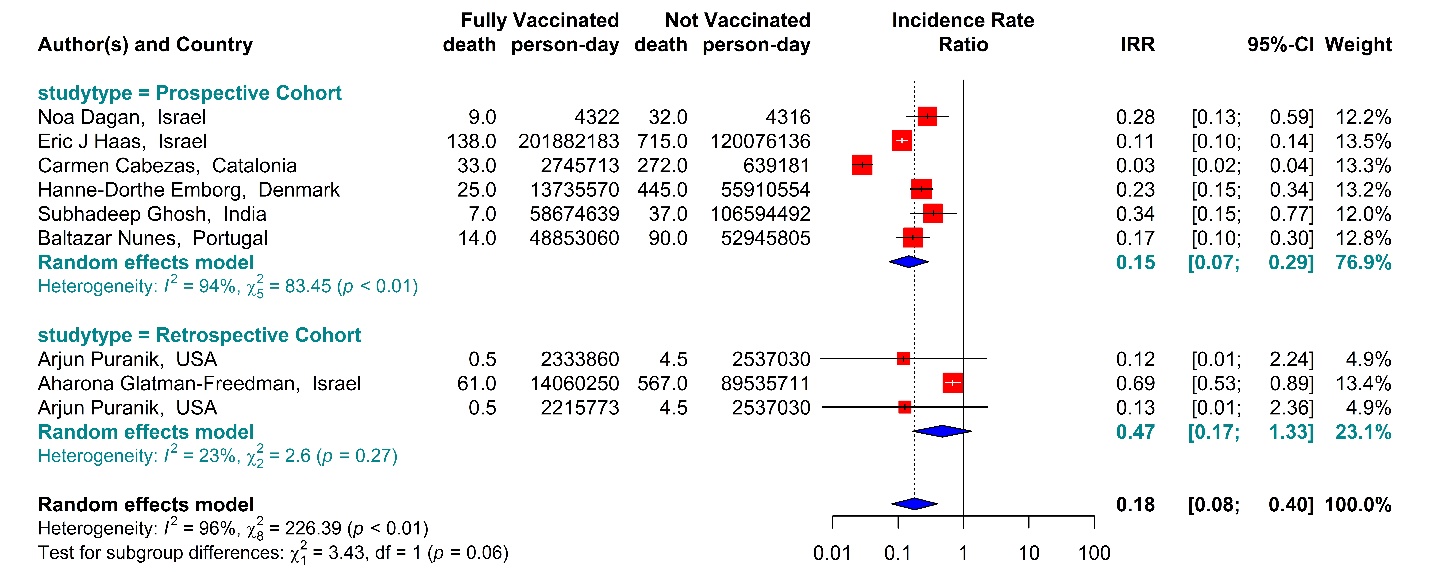


Figure 12. Full effectiveness of vaccines against COVID-19-related mortality using Incidence Rate Ratio by type of study

Table1- quality assessment in case-control studies.

| selection | | | | Comparability | Exposure | | |  |  |
| --- | --- | --- | --- | --- | --- | --- | --- | --- | --- |
| Case-control studies | *Case*  *definition* | *Representativeness of the cases* | *Selection of Controls* | *Definition of Controls* | *Control for most important factor and*  *Control for any additional factor* | *Ascertainment of exposure* | *Same method of ascertainment for cases and controls* | *Non-Response rate* | *score* |
| Jamie Lopez Bernal | a (1) | a (1) | a (1) | a (1) | a , b (2) | a (1) | a (1) | b (0) | 8 |
| Hannah Chung | a (1) | a (1) | a (1) | a (1) | a , b (2) | a (1) | a (1) | b (0) | 8 |
| M.G. Thompson | a (1) | a (1) | a(1) | a (1) | a , b (2) | a(1) | a (1) | b (0) | 8 |
| Jamie Lopez Bernal | a (1) | a (1) | a (1) | a(1) | a , b (2) | a(1) | a (1) | b (0) | 8 |
| T. Pilishvili | a (1) | a (1) | a (1) | a(1) | a , b (2) | a(1) | a(1) | a (1) | 9 |
| Tamara Pilishvili | a (1) | a (1) | a (1) | a(1) | a(1) | a(1) | a(1) | b(0) | 7 |
| Ioannis Baltas | a (1) | a (1) | a (1) | a (1) | a , b (2) | a(1) | a(1) | b(0) | 8 |
| Alyson M. Cavanaugh | a (1) | a (1) | a (1) | b (0) | a(1) | a(1) | a(1) | a(1) | 7 |
| Sara Carazo | a (1) | a (1) | a (1) | a (1) | a (1) | a (1) | a (1) | b(0) | 7 |
| Kristin L. Andrejko | b(0) | a (1) | a (1) | a (1) | a (1) | a (1) | a (1) | a (1) | 7 |
| Wesley H. Self | a (1) | a (1) | a (1) | a (1) | a(1) | c(0) | a (1) | a (1) | 7 |

Table2- quality assessment in cohort studies.

| selection | | | | Comparability | outcome | | |  |  |
| --- | --- | --- | --- | --- | --- | --- | --- | --- | --- |
| Cohort studies | Representativeness of the exposed cohort | Selection of the non-exposed cohort | Ascertainment of exposure | Outcome was not present as baseline | *Control for most important factor and*  *Control for any additional factor* | Assessment of outcome | Adequate follow-up period for outcome | Adequacy of follow up of cohorts | score |
| Ben Glampson | a(1) | a (1) | a (1) | a(1) | a , b (2) | a(1) | a (1) | a (1) | 9 |
| Carmen Cabezas | a(1) | a (1) | a (1) | a(1) | a , b (2) | b (1) | a (1) | a (1) | 9 |
| Aharona Glatman | a(1) | a (1) | a (1) | a(1) | a , b (2) | b (1) | a (1) | a (1) | 9 |
| Galia Zacay | a(1) | a(1) | a(1) | a (1) | a , b (2) | b (1) | a (1) | a (1) | 9 |
| Tariq Azamgarhi | a(1) | a(1) | a(1) | a(1) | a , b (2) | a(1) | a (1) | a (1) | 9 |
| Nathanael Fillmore | a(1) | a(1) | a(1) | a(1) | a(1) | b(1) | a(1) | d(0) | 7 |
| Victoria Jane Hall | a(1) | a(1) | c(0) | a(1) | a , b (2) | b(1) | a (1) | a (1) | 8 |
| Tara C. Bouton | a(1) | a(1) | a(1) | a(1) | a , b (2) | a(1) | a (1) | a (1) | 9 |
| Subhadeep Ghosh | b(1) | a(1) | a(1) | a(1) | a , b (2) | b(1) | a (1) | a (1) | 9 |
| Noa Dagan | a(1) | a(1) | a(1) | a(1) | a , b (2) | a(1) | a (1) | a (1) | 9 |
| Hall V FFPH | b(1) | a(1) | a(1) | a(1) | a , b (2) | b(1) | a (1) | a (1) | 9 |
| Saurabh Bobdey | c(0) | a(1) | a(1) | a(1) | a(1) | a(1) | a (1) | a (1) | 7 |
| Iván Martínez-Baz | c(0) | a(1) | a(1) | a(1) | a , b (2) | a(1) | a (1) | a (1) | 8 |
| Massimo Fabiani | b(1) | a(1) | a(1) | a(1) | a(1) | a(1) | a (1) | a (1) | 8 |
| Amadea Britton | b(1) | a(1) | a(1) | a(1) | a , b (2) | b(1) | a (1) | a (1) | 9 |
| Eric J Haas | a(1) | a(1) | a(1) | a(1) | a , b (2) | b(1) | a (1) | a (1) | 9 |
| Ping Ye, DNP | b(1) | a(1) | a(1) | a(1) | a(1) | b(1) | a (1) | a (1) | 8 |
| Maria Elena Flacco | a(1) | a(1) | a(1) | a(1) | a (1) | a(1) | a (1) | a (1) | 8 |
| Mark G. Thompson | b(1) | a(1) | c(0) | a(1) | a (1) | b(1) | a (1) | a (1) | 7 |
| Baltazar Nunes | a(1) | a(1) | a(1) | a(1) | a , b (2) | b(1) | a (1) | a (1) | 9 |
| Adeel A. Butt | a(1) | a(1) | a(1) | a(1) | a , b (2) | a (1) | a (1) | a (1) | 9 |
| M.G. Thompson | b(1) | a(1) | a(1) | a(1) | a , b (2) | a(1) | a(1) | a(1) | 9 |
| Iván Martínez-Baz | c(0) | a(1) | a(1) | a(1) | a(1) | b(1) | a(1) | a(1) | 7 |
| Sarah E. Waldman | c(0) | a(1) | a(1) | a(1) | a(1) | b(1) | a(1) | a(1) | 7 |
| Aleena Issac | b(1) | a(1) | a(1) | a(1) | a , b (2) | b(1) | a(1) | a(1) | 9 |
| Jennifer B. Griffin | a(1) | a(1) | a(1) | a(1) | a(1) | b(1) | a(1) | a(1) | 8 |
| Madhumita Shrotri | b(1) | a(1) | a(1) | a(1) | a , b (2) | b(1) | a(1) | a(1) | 9 |
| Anoop S V Shah | b(1) | a(1) | a(1) | a(1) | a , b (2) | b(1) | a(1) | a(1) | 9 |
| Ashley Fowlkes | b(1) | a(1) | a(1) | a(1) | a(1) | b(1) | a(1) | a(1) | 8 |
| Eli S. Rosenberg | a(1) | a(1) | a(1) | a(1) | a(1) | b(1) | a(1) | a(1) | 8 |
| Aaron J. Tande | a(1) | a(1) | a(1) | a(1) | a , b (2) | b(1) | a(1) | a(1) | 9 |
| Colin Pawlowski | a(1) | a(1) | a(1) | a(1) | a , b (2) | b(1) | a(1) | a(1) | 9 |
| Gili Regev-Yochay | b(1) | a(1) | a(1) | a(1) | a , b (2) | b(1) | a(1) | a(1) | 9 |
| Arjun Puranik | a(1) | a(1) | a(1) | a(1) | a , b (2) | b(1) | a(1) | a(1) | 9 |
| Mark A. Katz | b(1) | a(1) | a(1) | a(1) | a(1) | b(1) | a(1) | a(1) | 8 |
| Jamie Lopez Bernal | a(1) | a(1) | a(1) | a(1) | a(1) | b(1) | a(1) | a(1) | 8 |
| Ida Rask Moustsen-Helms | b(1) | a(1) | a(1) | a(1) | a , b (2) | b(1) | a(1) | a(1) | 9 |
| Hanne-Dorthe Emborg | b(1) | a(1) | a(1) | a(1) | a , b (2) | b(1) | a(1) | a(1) | 9 |
| Jonas Björk | a(1) | a(1) | a(1) | a(1) | a(1) | b(1) | a(1) | a(1) | 8 |
| Susana Monge | b(1) | a(1) | a(1) | a(1) | a(1) | b(1) | a(1) | a(1) | 8 |
| Yoel Angel | b(1) | a(1) | a(1) | a(1) | a(1) | b(1) | a(1) | a(1) | 8 |
| Sara Y. Tartof | a(1) | a(1) | a(1) | a(1) | a , b (2) | b(1) | a(1) | a(1) | 9 |
| Peter Nordstrom | a(1) | a(1) | a(1) | a(1) | a(1) | b(1) | a(1) | a(1) | 8 |
