# Supplementary material for: The effectiveness of COVID-19 vaccines in reducing the incidence, hospitalization, and mortality from COVID-19: A systematic review and meta-analysis
Source: Front Public Health. 2022 Aug 26;10:873596. doi: 10.3389/fpubh.2022.873596 (PMC9459165; doi:10.3389/fpubh.2022.873596)
Supplement: Supplementary file 2 [file Data_Sheet_2.docx]

**Supplementary.2**

**Sensitivity Analysis:**


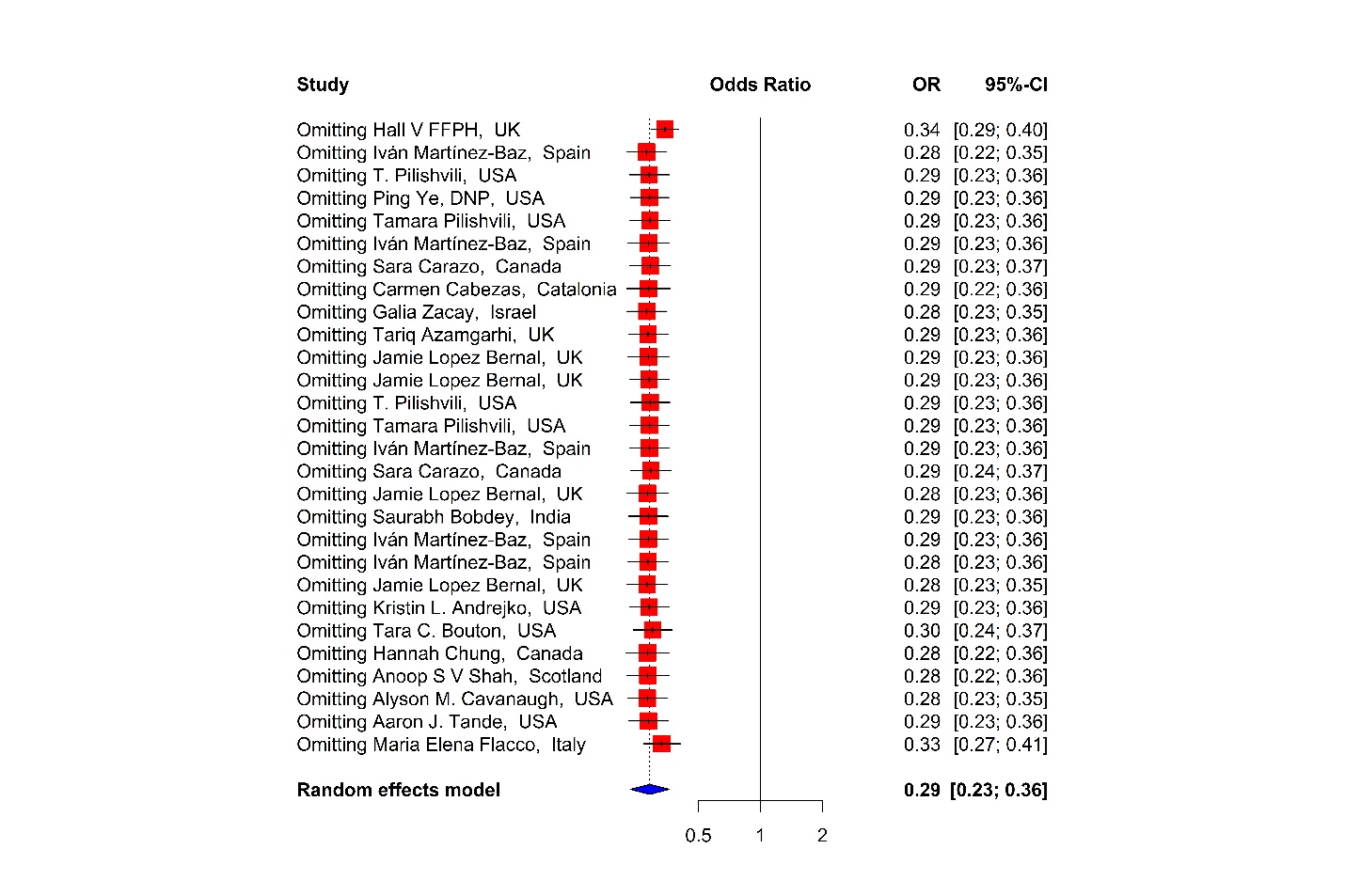


**Figure 1.** Sensitivity analysis for Partial vaccinated effectiveness of vaccines against SARS-COV 2 infection.


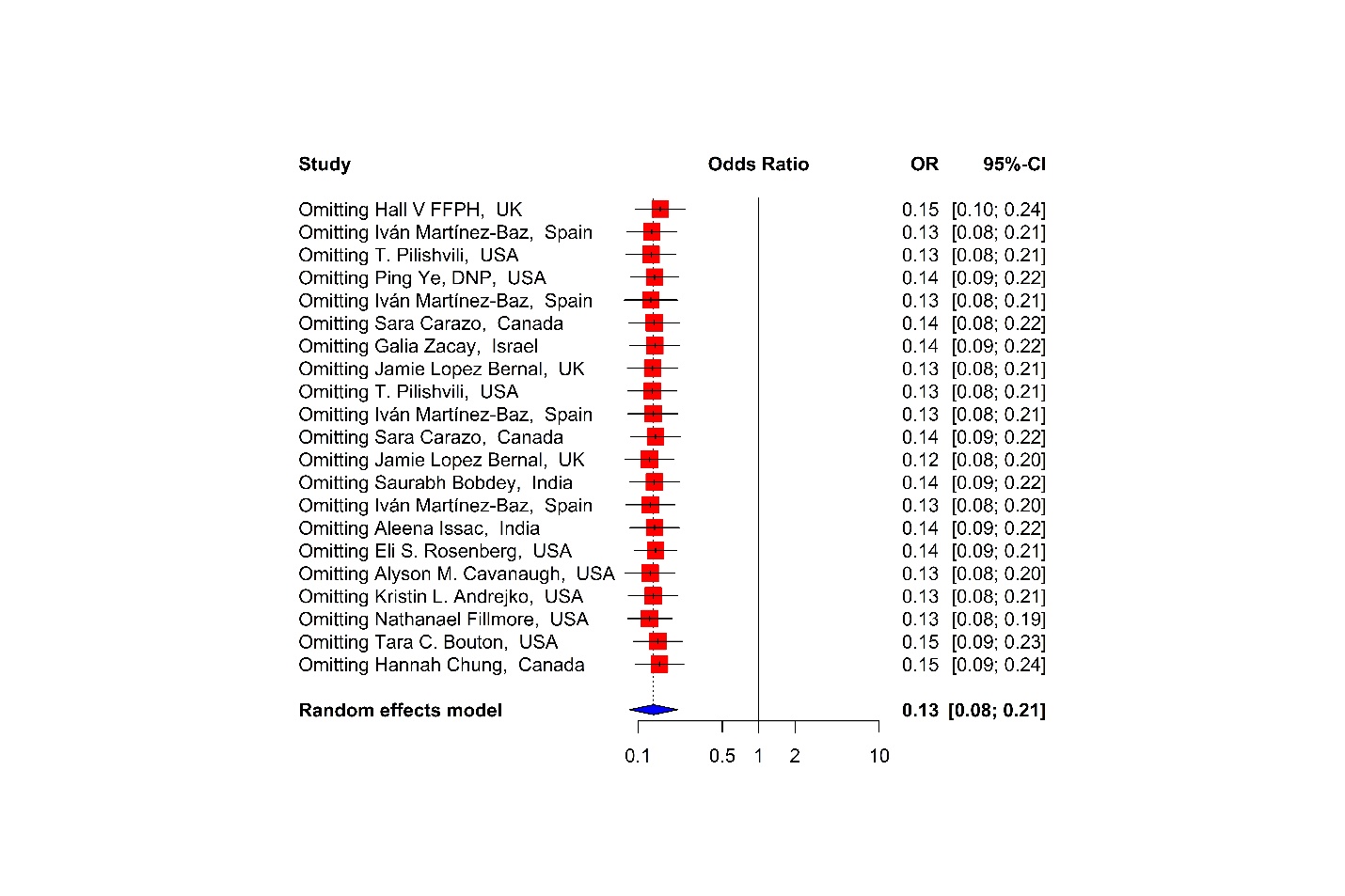


**Figure 2.** Sensitivity analysis for Full vaccinated effectiveness of vaccines against SARS-COV 2 infection.


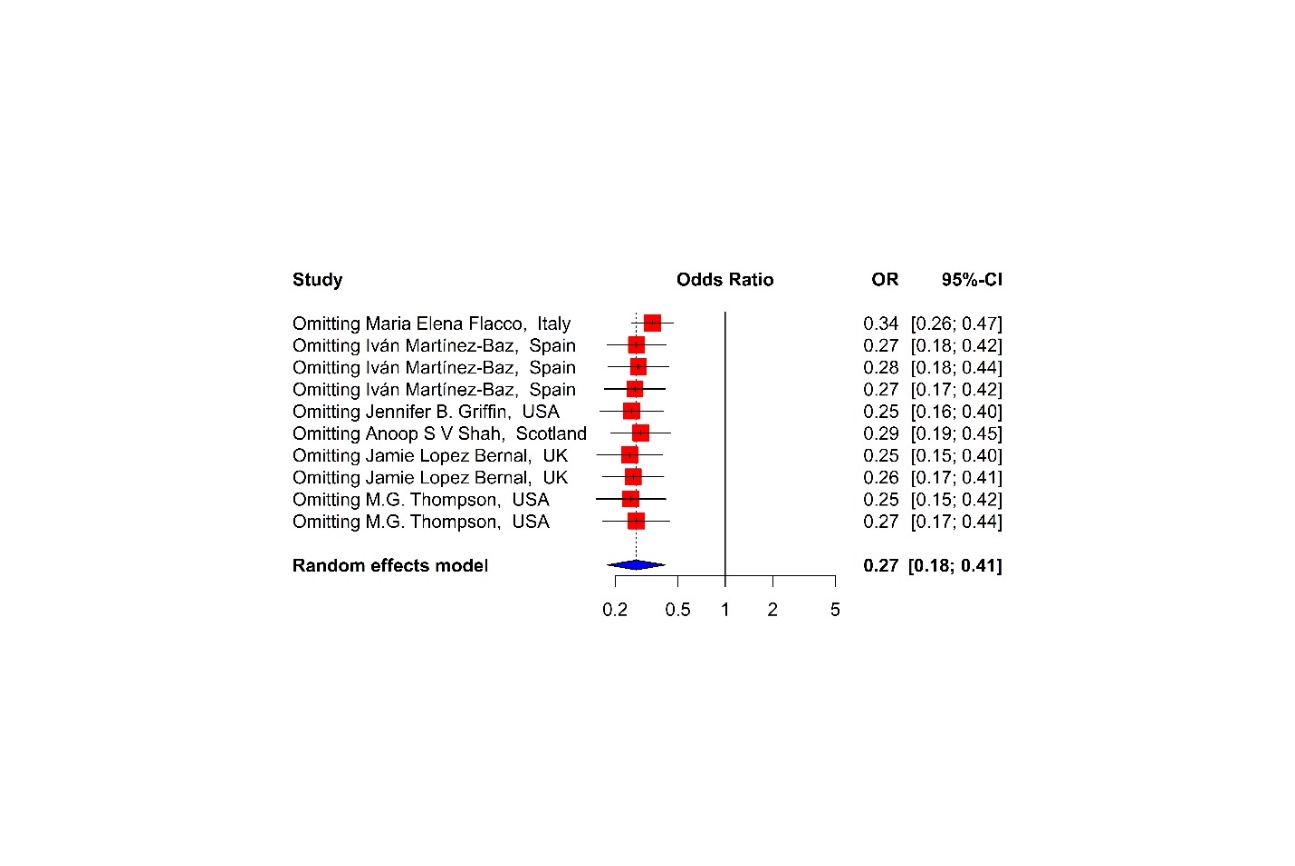


**Figure 3.** Sensitivity analysis for Partial effectiveness of vaccines against COVID-19-related hospitalization.


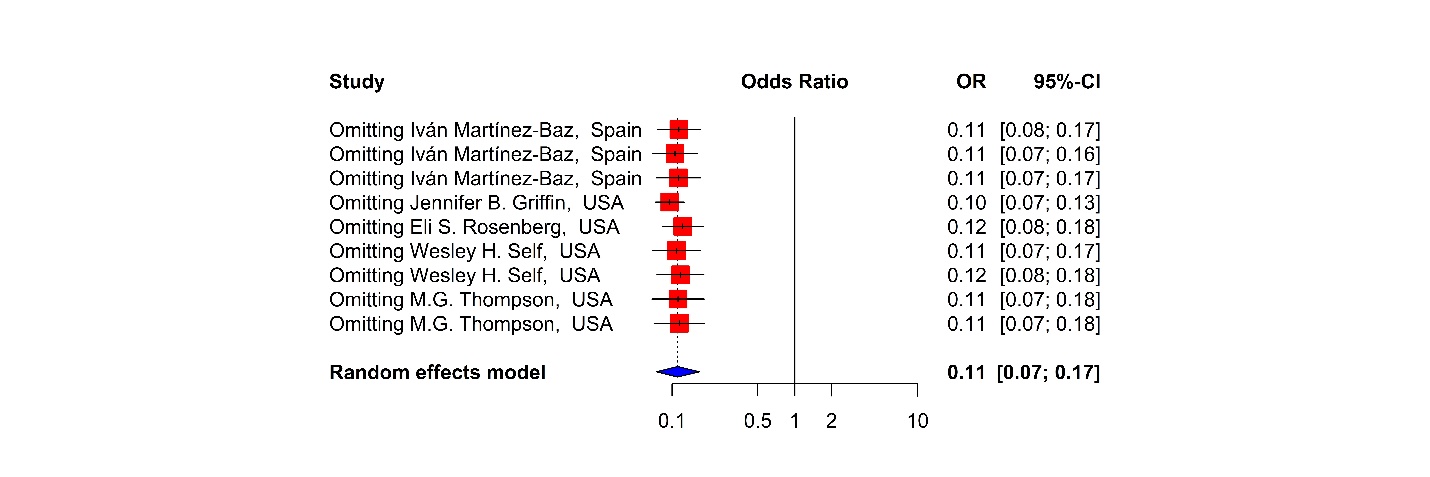


**Figure 4.** Sensitivity analysis for Full effectiveness of vaccines against COVID-19-related hospitalization.


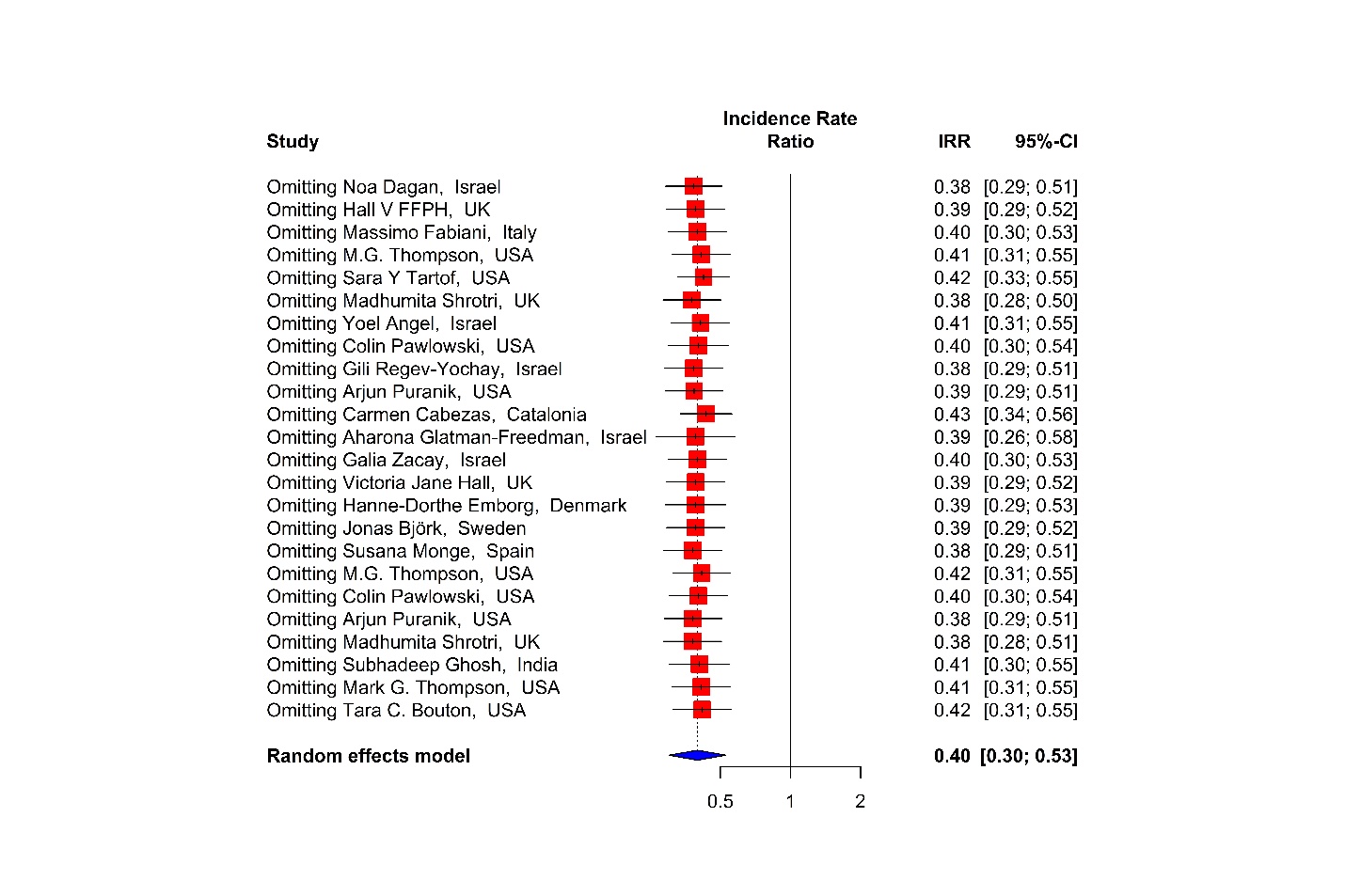


**Figure 5.** Sensitivity analysis for partial Effectiveness of vaccines against SARS-COV 2 infection using Incidence rate ratio.


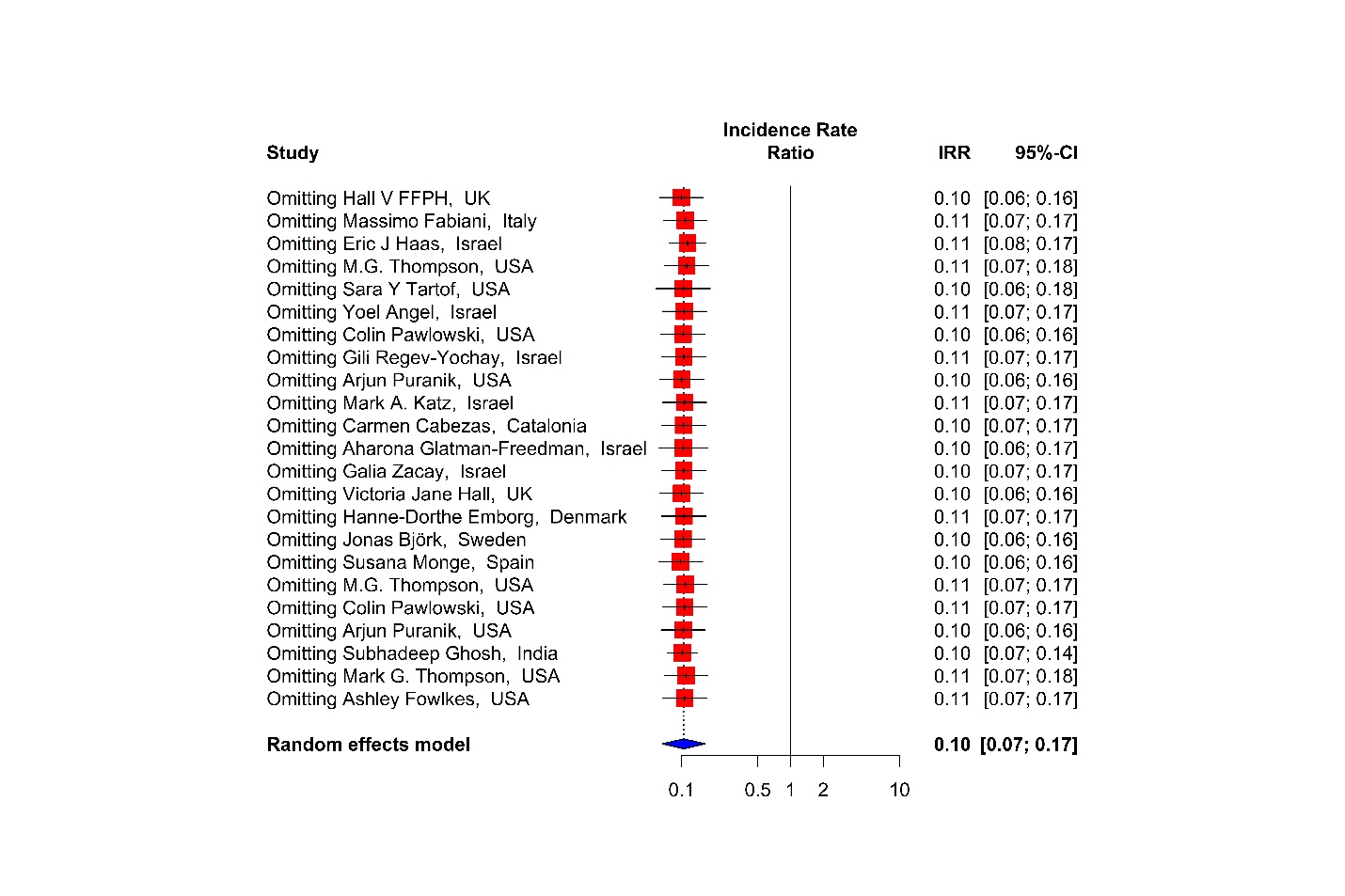


**Figure 6.** Sensitivity analysis for Full Effectiveness of vaccines against SARS-COV 2 infection using Incidence rate ratio.


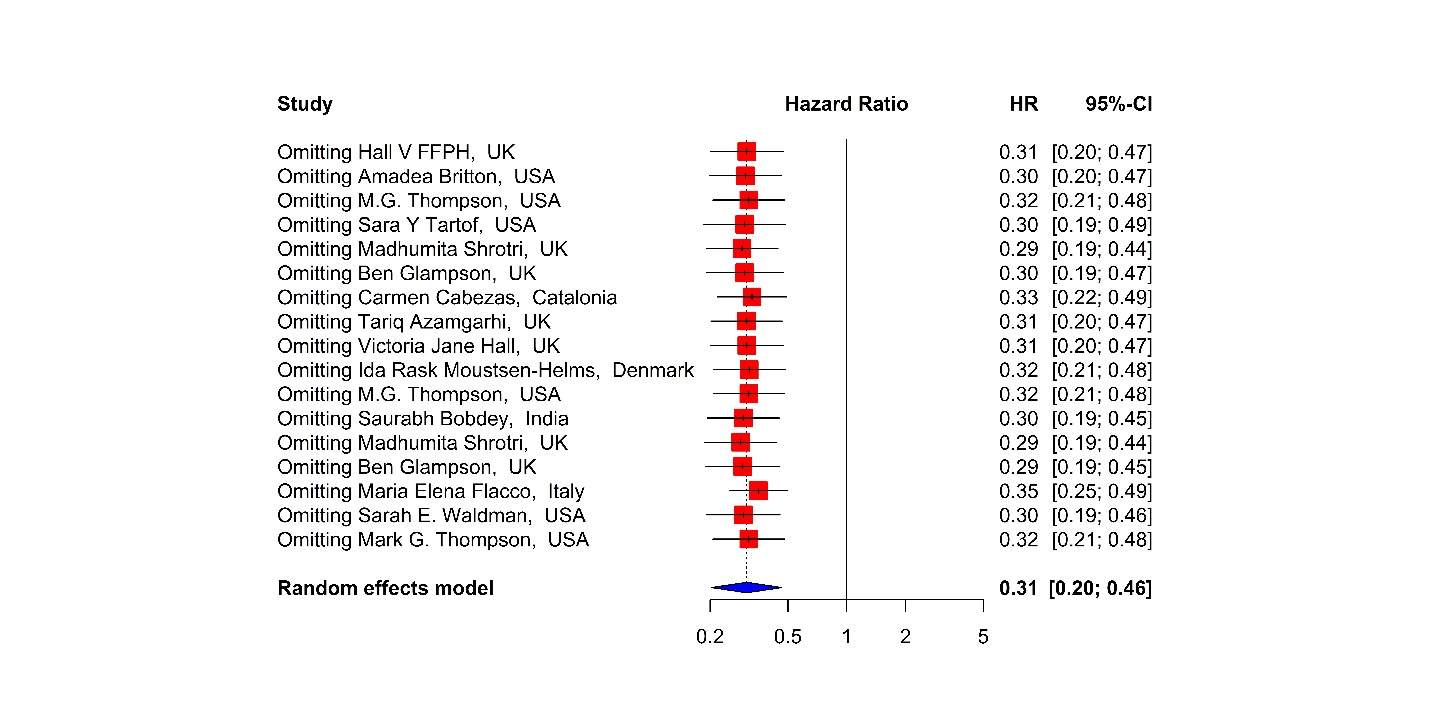


**Figure 7.** Sensitivity analysis for Partial Effectiveness of vaccines against SARS-COV 2 infection using Hazard ratio.


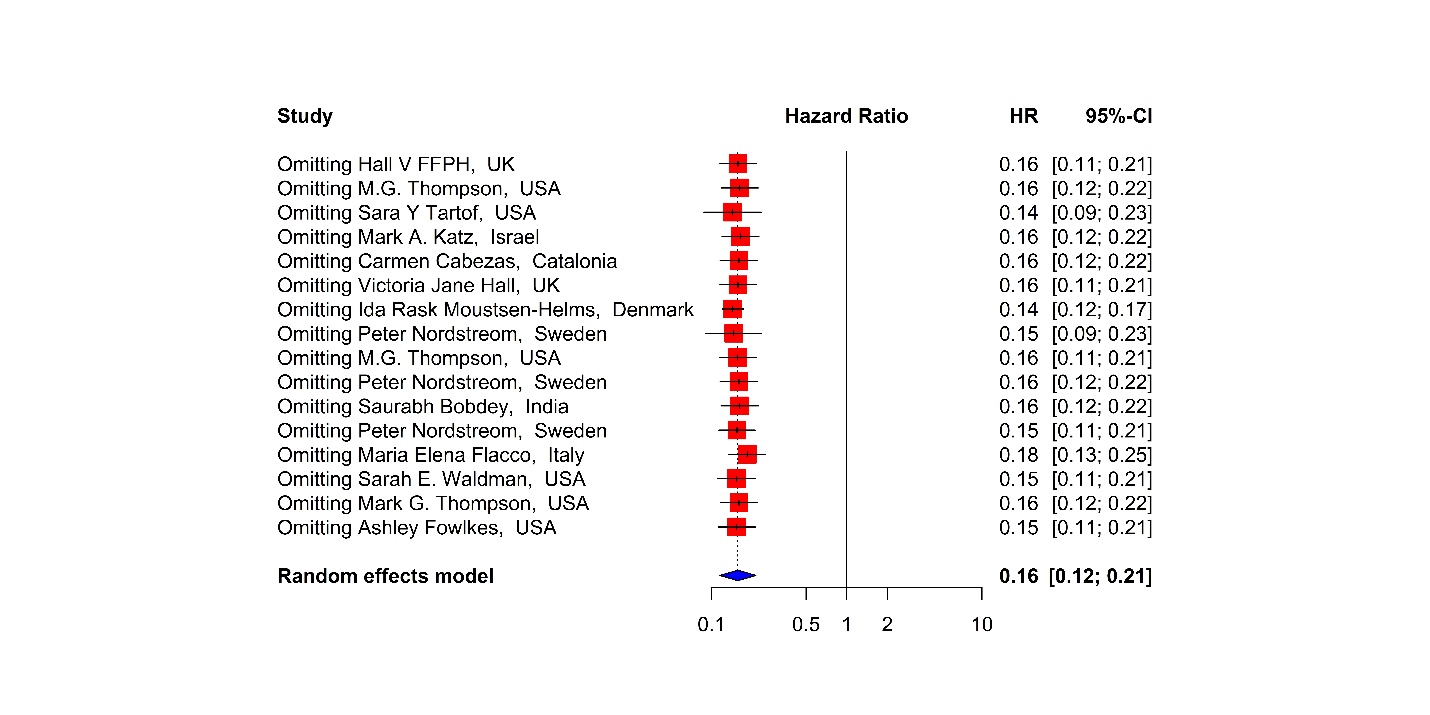


**Figure 8.** Sensitivity analysis for Full Effectiveness of vaccines against SARS-COV 2 infection using Hazard ratio.


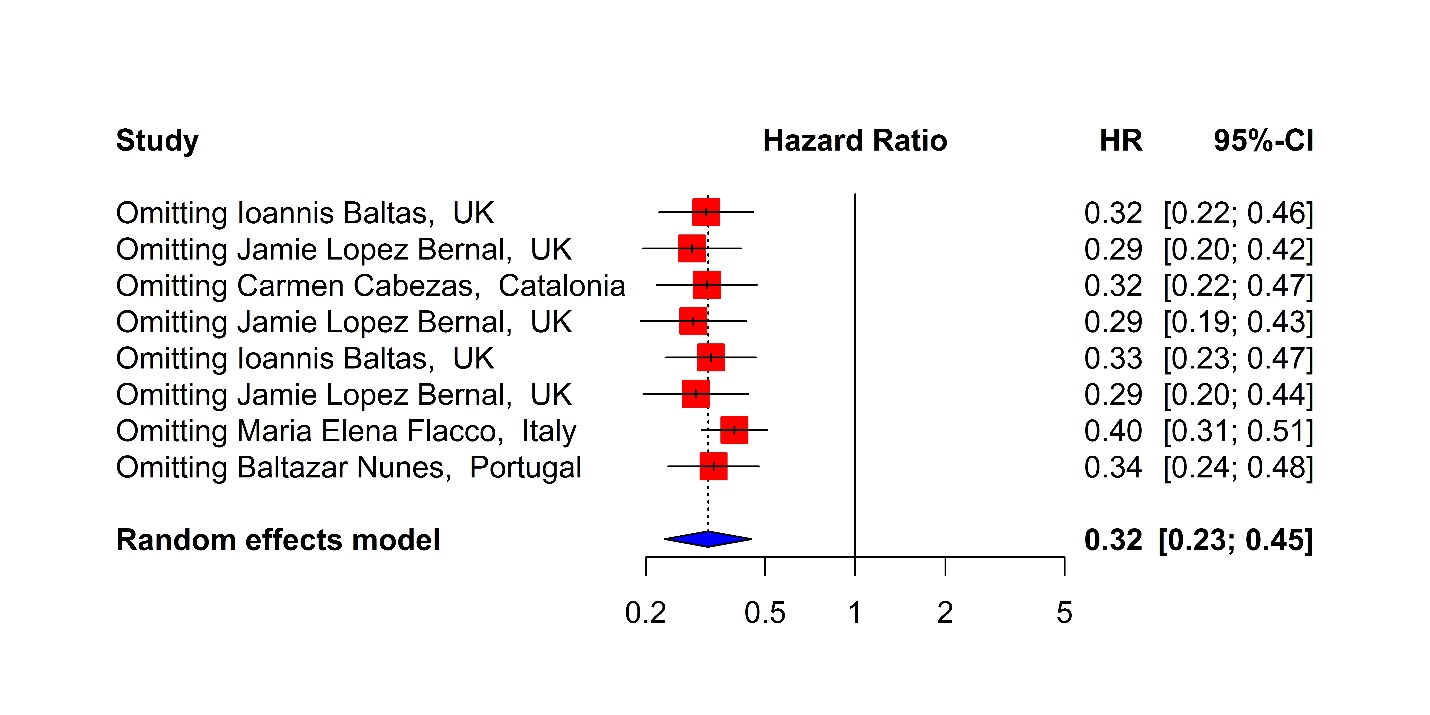


**Figure 9.** Partial Effectiveness of vaccines against COVID-19-related mortality using Hazard Ratio.


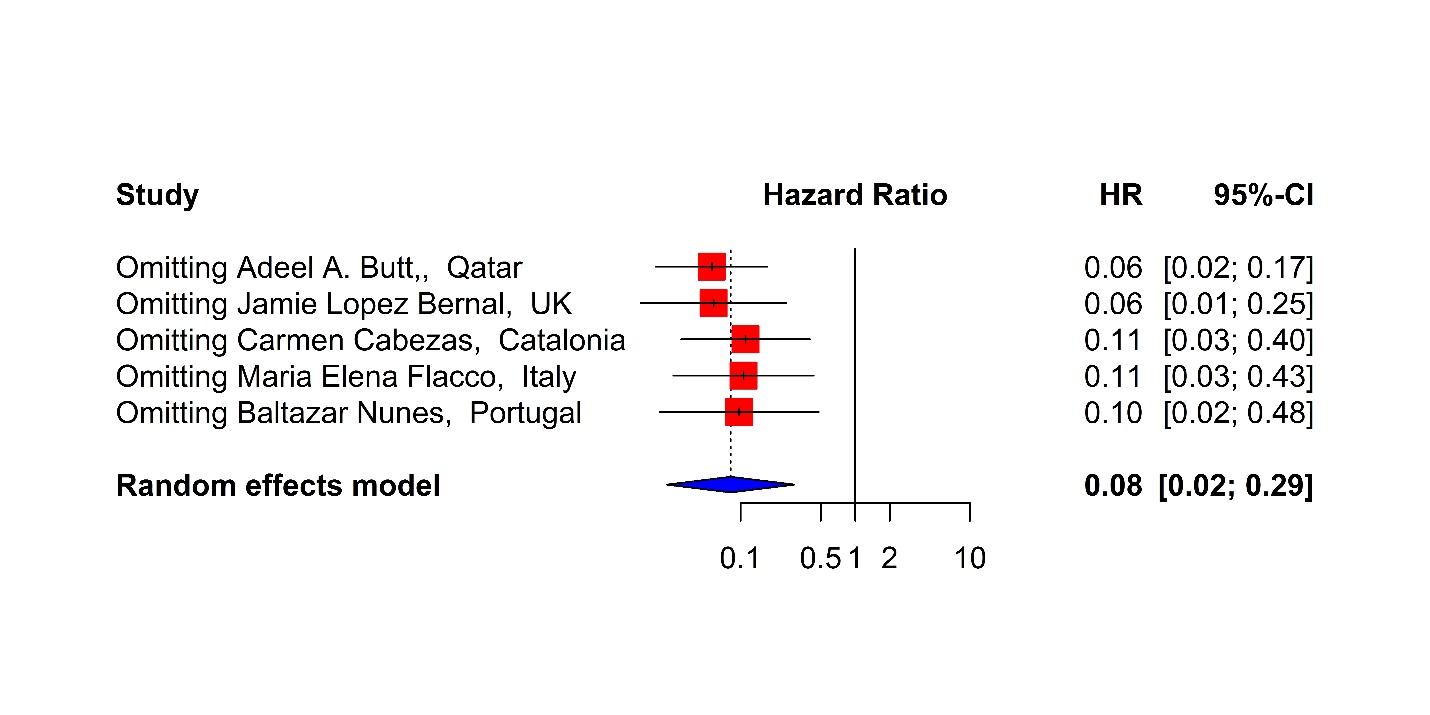


**Figure 10.** Full Effectiveness of vaccines against COVID-19-related mortality using Hazard Ratio.


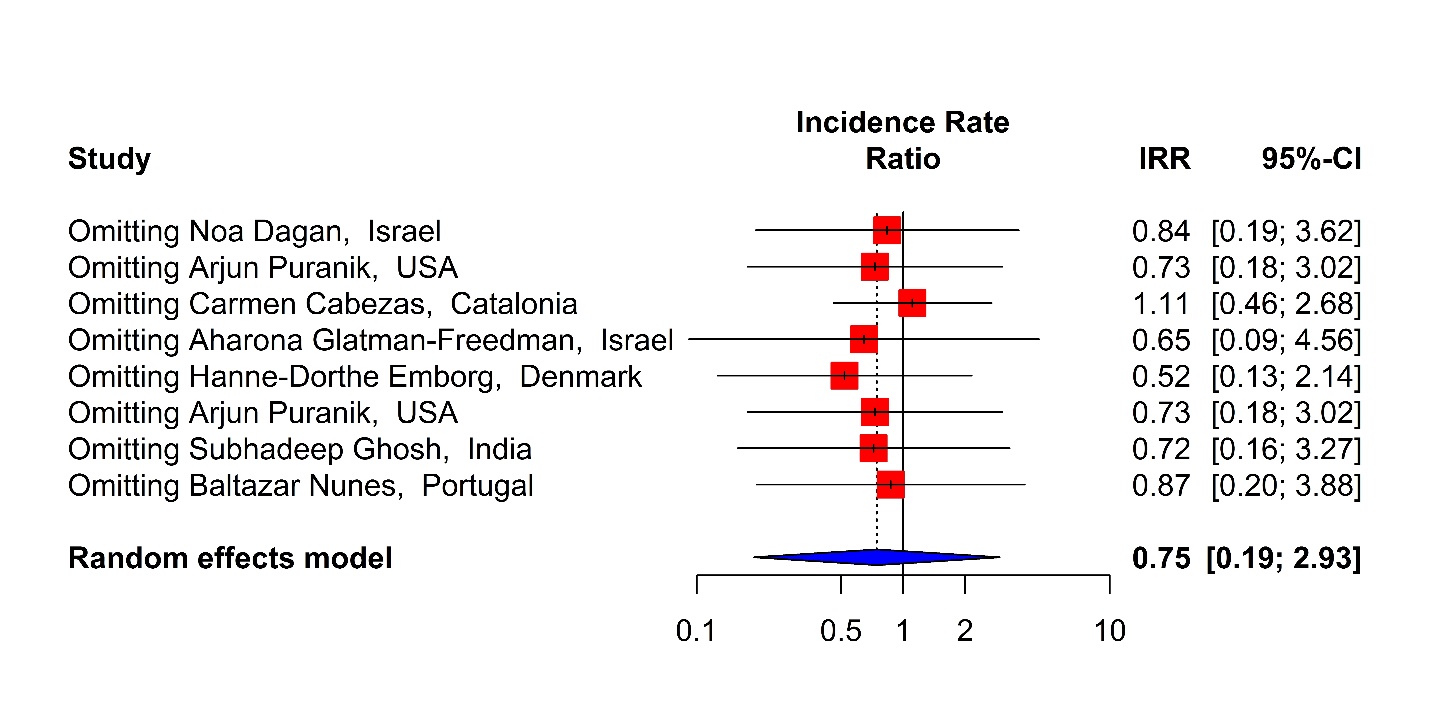


**Figure 11**. Partial effectiveness of vaccines against COVID-19-related mortality using Incidence Rate Ratio.


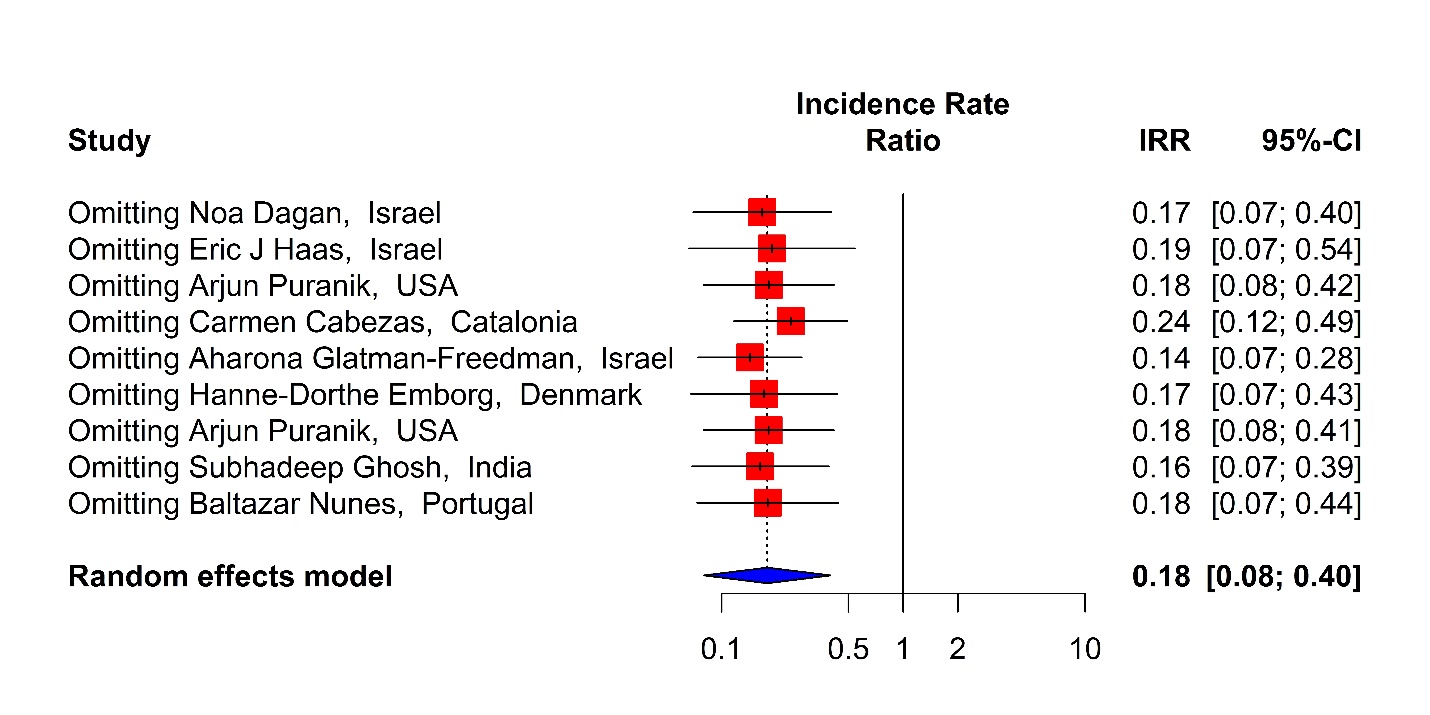


**Figure 12**. Full effectiveness of vaccines against COVID-19-related mortality using Incidence Rate Ratio.

**Publication Bias:**


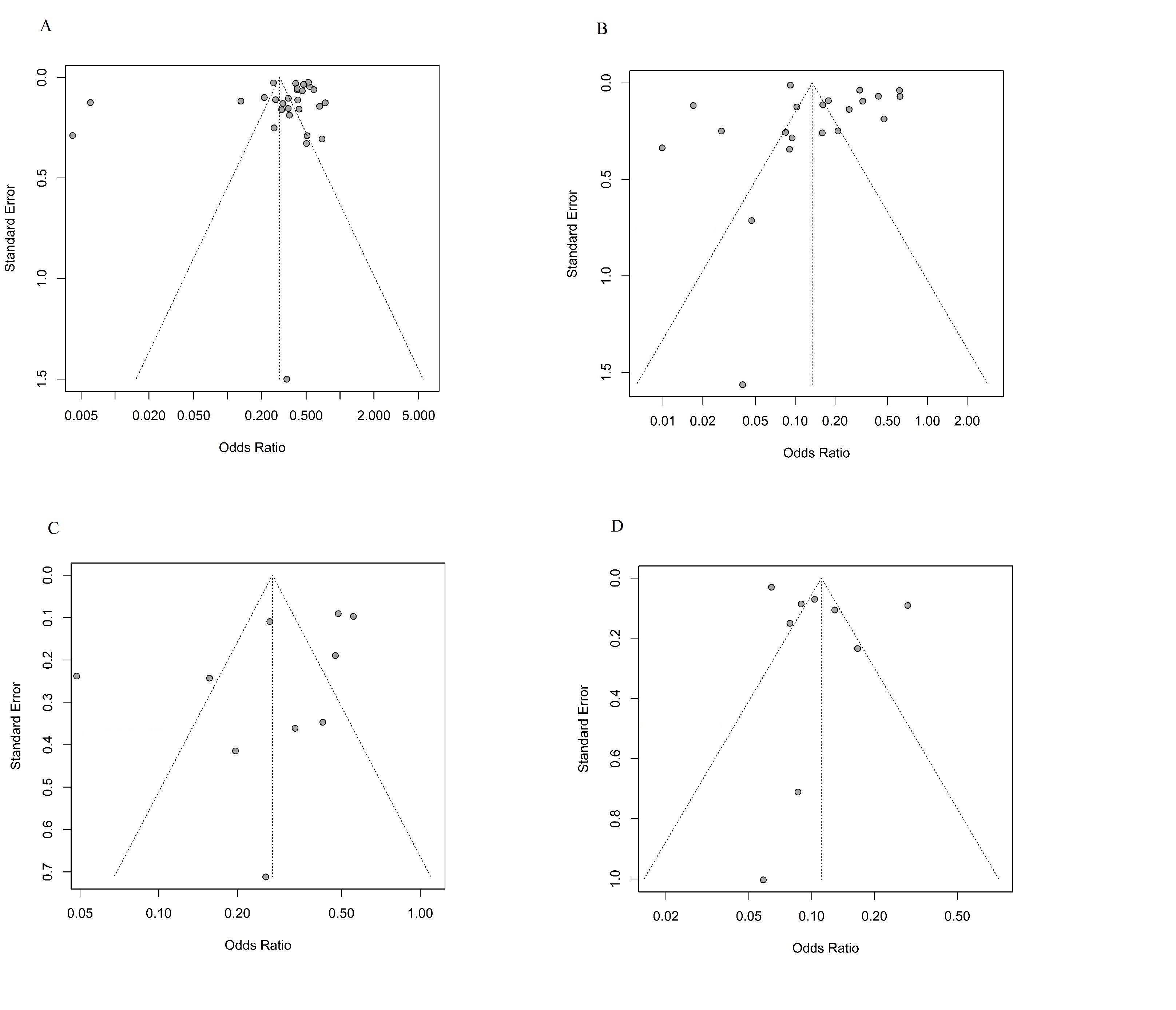


**Figure 13.** Funnel plots not indicating publication bias for **A**; Partial vaccinated effectiveness vaccines against SARS-COV 2 infection (Eggers' test P value= 0.180), **B**; Full vaccinated effectiveness vaccines against SARS-COV 2 infection (Eggers' test P value= 0.204), **C**; Partial effectiveness of vaccines against COVID-19-related hospitalization (Eggers' test P value= 0.178), **D**; Full effectiveness of vaccines against COVID-19-related hospitalization (Eggers' test P value= 0.173).


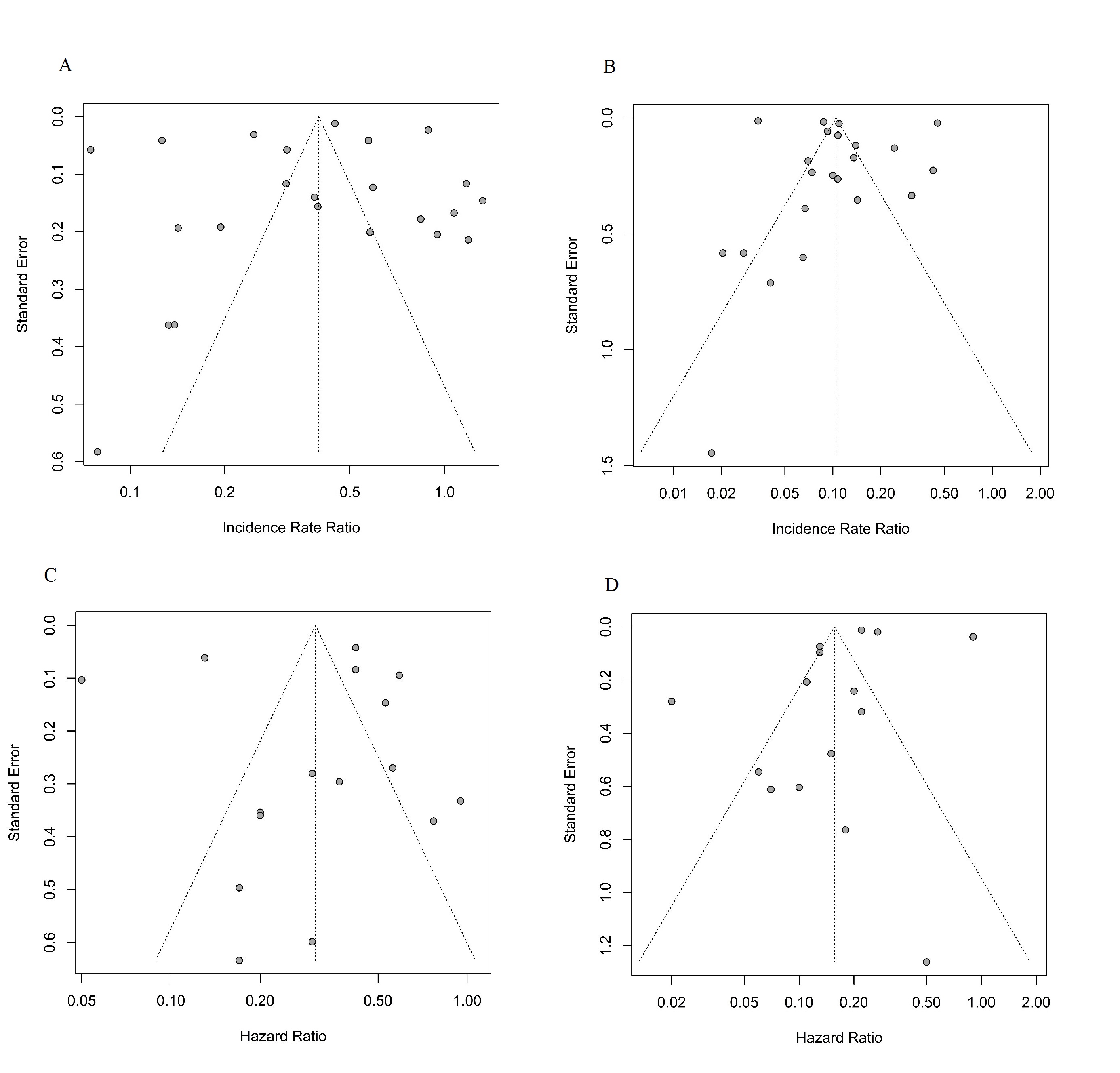


**Figure 14.** Funnel plots not indicating publication bias for **A**; Partial effectiveness of vaccines against SARS-COV 2 infection using Incidence Rate Ratio (Eggers' test P value= 0.601), **B**; Full effectiveness of vaccines against SARS-COV 2 infection using Incidence Rate Ratio (Eggers' test P value= 0.406), **C**; Partial effectiveness of vaccines against SARS-COV 2 infection using Hazard Ratio (Eggers' test P value= 0.974), **D**; Full effectiveness of vaccines against SARS-COV 2 infection using Hazard Ratio (Eggers' test P value= 0.786).


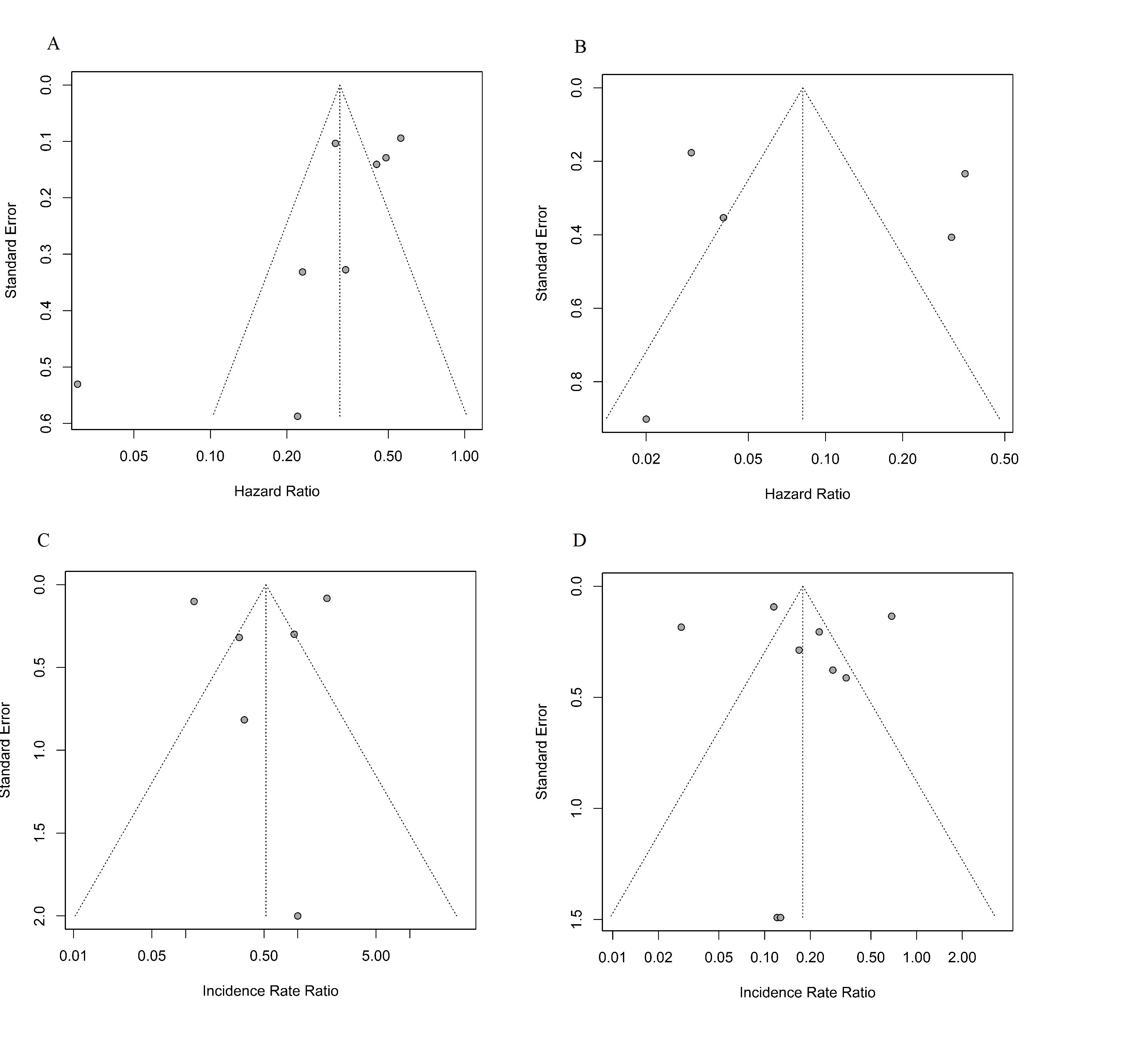


**Figure 15.** Funnel plots not indicating publication bias for **A**; Partial effectiveness of vaccines against COVID-19-related mortality using Hazard ratio. (Eggers' test P value= 0.0917), **B**; Full effectiveness of vaccines against COVID-19-related mortality using Hazard ratio. (Eggers' test P value= 0.796), **C**; Partial effectiveness of vaccines against COVID-19-related mortality using Incidence Rate Ratio (Eggers' test P value= 0.842), **D**; Full effectiveness of vaccines against COVID-19-related mortality using Incidence Rate Ratio (Eggers' test P value= 0.883).
